# *N*-Acyloxymethyl-phthalimides deliver genotoxic formaldehyde to human cells†

**DOI:** 10.1039/d3sc02867d

**Published:** 2023-09-15

**Authors:** Vicki L. Emms, Liam A. Lewis, Lilla Beja, Natasha F. A. Bulman, Elisabete Pires, Frederick W. Muskett, James S. O. McCullagh, Lonnie. P. Swift, Peter J. McHugh, Richard J. Hopkinson

**Affiliations:** a Institute for Structural and Chemical Biology, School of Chemistry, University of Leicester Henry Wellcome Building Lancaster Road Leicester LE1 7RH UK richard.hopkinson@leicester.ac.uk; b Department of Chemistry, University of Oxford, Chemistry Research Laboratory 12 Mansfield Road Oxford OX1 3TA UK; c Institute for Structural and Chemical Biology, Department of Molecular and Cell Biology, University of Leicester Henry Wellcome Building Lancaster Road Leicester LE1 7RH UK; d Department of Oncology, MRC Weatherall Institute of Molecular Medicine, University of Oxford, John Radcliffe Hospital Headington Oxford OX3 9DS UK lonnie.swift@imm.ox.ac.uk peter.mchugh@imm.ox.ac.uk

## Abstract

Formaldehyde is a pollutant and human metabolite that is toxic at high concentrations. Biological studies on formaldehyde are hindered by its high reactivity and volatility, which make it challenging to deliver quantitatively to cells. Here, we describe the development and validation of a set of *N*-acyloxymethyl-phthalimides as cell-relevant formaldehyde delivery agents. These esterase-sensitive compounds were similarly or less inhibitory to human cancer cell growth than free formaldehyde but the lead compound increased intracellular formaldehyde concentrations, increased cellular levels of thymidine derivatives (implying increased formaldehyde-mediated carbon metabolism), induced formation of cellular DNA-protein cross-links and induced cell death in pancreatic cancer cells. Overall, our *N*-acyloxymethyl-phthalimides and control compounds provide an accessible and broadly applicable chemical toolkit for formaldehyde biological research and have potential as cancer therapeutics.

## Abstract 2

*N*-Acyloxymethyl-phthalimides are esterase-sensitive chemical tools that enable controllable delivery of formaldehyde to human cells.

## Graphical abstract

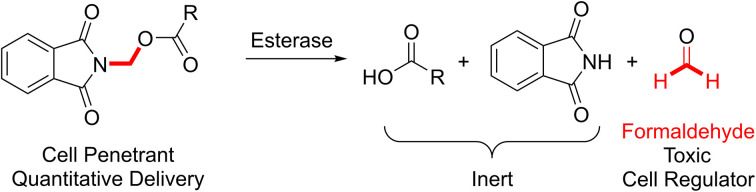

## Introduction

Formaldehyde (HCHO), the simplest aldehyde, is a human toxin and carcinogen.^1,2^ Acute exposure can lead to nausea, convulsions, renal failure and coma,^3^ whereas low-level chronic exposure correlates with the onset of multiple cancers.^4^ While exposure can occur from environmental sources,^5–7^ HCHO is also a human metabolite produced during tetrahydrofolate degradation^8,9^ and enzymatic demethylation reactions occurring on proteins and nucleic acids.^10–15^ This endogenous HCHO production suggests that all human cells are continuously exposed to HCHO and that its health implications are context- and concentration-dependent. While too much HCHO can be toxic, particularly in cells lacking a functional Fanconi anaemia DNA repair pathway,^16^ HCHO also plays roles in healthy cells, including as a carbon source in nucleotide biosynthesis.^8^ This suggests HCHO has other as-yet unknown functions with health and disease implications, although the mechanisms underpinning these functions remain unclear.

From a chemical perspective, HCHO is highly reactive with a variety of biomolecules, forming multiple hydroxymethyl and cross-linked adducts of varying stabilities.^17–25^ As is the case for adducts derived from other reactive biomolecules such as nitric oxide, hydrogen sulfide or methylglyoxal, these HCHO-derived adducts are likely to be responsible for many of HCHO's biological functions; however, their detection in cells is very challenging due to their low abundance and often short life-times. Efforts to detect HCHO in cells would be greatly assisted by increasing intracellular HCHO concentrations, although current methods to do this have limitations. Adding free HCHO to tissue culture cell media is compromised by HCHO's volatility (leading to evaporation from the media), polymerisation and/or disproportionation in water, and its reactions with other components in the media such as amino acids.^23^ Using high levels (*i.e.* millimolar concentrations or higher) of HCHO is also limited by the potential for cell fixing, while lower concentrations can lead to localised fixative effects.^26^ Genetic down-regulation of HCHO metabolism can impart genotoxicity in DNA repair-deficient cellular and animal models, which is almost certainly due to elevated HCHO concentrations.^16^ However, HCHO-metabolising enzymes are also involved in the metabolism of nitric oxide and other aldehydes.^27,28^ Consequently, these genetic analyses might not specifically or uniquely reveal HCHO's biological functions. HCHO-releasing small molecules also have potential in biological experiments. *N*-Hydroxymethylated compounds release HCHO in water and are common additives in cosmetics,^29^ while hexamethylenetetramine is a water-sensitive HCHO releaser that is used to treat urinary tract infections.^30^*N*-Acyloxymethyl group-containing pro-drugs, which release HCHO after enzyme-catalysed hydrolysis, can give better control of HCHO release due to their stability in water. However, while many of these compounds have important clinical relevance, they additionally release other bioactive molecules, which complicates analysis of HCHO release.^31–42^ Another common limitation with HCHO releasers is their poor solubility, which restricts the amount of HCHO that can be delivered to cells (note, there is also potential for toxic aggregation/precipitation). Recently reported pioneering light-sensitive HCHO donors overcome water stability issues (being soluble in the micromolar range) and induce HCHO-mediated effects on wound healing. However, the requirement for photoirradiation to enable HCHO release suggests they will not be broadly applicable across all cellular assays.^43^

**Fig. 1:**
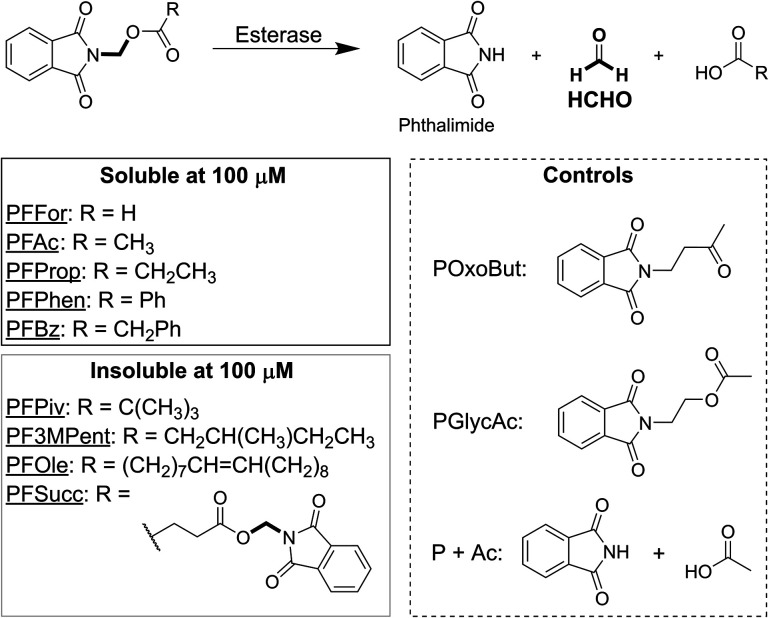
Structures of phthalimide-derived HCHO-releasing compounds. All compounds contain a phthalimide moiety connected *via* a methylene group to a carboxylate oxygen. Esterase-mediated hydrolysis of the resultant ester produces the free carboxylate, phthalimide and HCHO. PFFor, PFAc, PFProp, PFPhen and PFBz are all soluble at 100 μM in 10% v/v DMSO in water. POxoBut and PGlycAc (dashed box) are non-HCHO-releasing analogues of PFAc.

We are interested in developing methods and tools to help elucidate HCHO's biology. To this end, we have developed a series of HCHO-releasing small molecules that provide a broadly applicable method for controlling the delivery of HCHO to cells. These simple *N*-acyloxymethyl-phthalimides are sensitive to enzyme-catalysed hydrolysis (Fig. 1 and Scheme S1†) and are inspired by HCHO-releasing compounds used in cosmetics and by HCHO-releasing protecting groups found in pro-drugs. Using human U2OS (osteosarcoma) cells as a model, we show that *N*-acyloxymethyl-phthalimides inhibit cell growth *via* HCHO release, and that the lead compound PFAc increases intracellular HCHO levels and induces an increase in thymidine derivatives, suggesting an upregulation of HCHO-mediated folate metabolism. PFAc also potentially induces formation of DNA-protein cross-links (DPCs), while cytotoxicity studies with pancreatic cancer cell models suggested PFAc, and by extension other *N*-acyloxymethyl-phthalimides, have potential as cancer chemotherapeutics.

## Results and discussion

We initially designed and synthesised 9 putative HCHO-releasing small molecules for use in cellular assays (Fig. 1). All compounds contain phthalimide and ester groups connected by an *N*,*O*-linked methylene, and were synthesised in one step from (*N*-bromomethyl)-phthalimide and the appropriate carboxylic acid under basic conditions. Reactions were completed within a few hours at room temperature. In addition, two control compounds were synthesised (Fig. 1 and Scheme S1†); POxoBut is a steric homologue of the acetate- and HCHO-releasing PFAc but lacks the ester group (thus blocking esterase sensitivity), while PGlycAc mimics acetate release but does not fragment to phthalimide and HCHO, instead forming (*N*-hydroxyethyl)-phthalimide.

### *N-*Acyloxymethyl-phthalimides are HCHO-releasing agents sensitive to enzymatic hydrolysis

The solubility of the compounds was first tested. Each compound was solubilised at 100 mM concentration in DMSO, before aliquots were taken from each stock and diluted to 100 μM with water (*i.e.* the highest concentration to be used in cell growth inhibition experiments). Importantly, five of the compounds, PFFor, PFAc, PFProp, PFPhen and PFBz, were soluble at this concentration, which suggests they can deliver high levels of HCHO to cells (Fig. 1). Four of the compounds, PF3MPent, PFPiv, PFSucc, and PFOle, were insoluble at 100 μM and were therefore excluded from subsequent experiments.

The stabilities of the soluble compounds were also tested in Dulbecco Modified Eagle Medium media with 10% v/v heat-inactivated foetal bovine serum (DMEM with FBS) at 37 °C. Concentrated samples of PFFor, PFAc, PFProp, PFPhen, PFBz, POxoBut and PGlycAc were incubated in DMEM with FBS in NMR tubes and monitored periodically over 7 hours by ^1^H NMR. Samples with PFPhen and PFBz could not be analysed due to their precipitation at 7 mM; however, analysis of the other samples did not indicate precipitation but revealed a reduction in the intensities of the ^1^H resonances corresponding to the phthalimide group, suggesting degradation of the compounds (percentage of starting compound remaining after two hours: PFAc = 44%, PFProp = 57%, PGlycAc = 83%, POxoBut = 61%, Fig. S1 and S2.† Note: signal overlap precluded quantification of PFFor). Importantly, however, addition of excess dimedone (a HCHO scavenger that forms a hydroxymethyl adduct amenable to ^1^H NMR analysis)^15^ after 24 hours revealed only low-level formation of HCHO in the samples with PFAc and PFProp (<10% relative to initial compound concentration), while no HCHO was detected in the samples with PGlycAc and POxoBut (Fig. S3†). With PFFor, ester hydrolysis was observed (Fig. S1B†), while addition of dimedone to this sample after 27 hours revealed a roughly 3-fold higher concentration of HCHO than with PFAc (Fig. S3†). Incubation of HCHO with DMEM with FBS for 24 hours followed by addition of dimedone revealed >10-fold higher HCHO concentration than that observed in the sample with PFAc (Fig. S3†). NMR characterisation of the PFAc- and PFProp-derived degradation products in DMEM with FBS was hindered by signal overlap. However, NMR and LC/MS analyses in either 100 mM sodium phosphate buffer in D_2_O pH 7.5, or in water and acetonitrile mixtures, revealed formation of the same products and enabled their assignment as *N*-acyloxymethyl-phthalamic acids (Fig. S4–S6 and Scheme S2†).

**Fig. 2:**
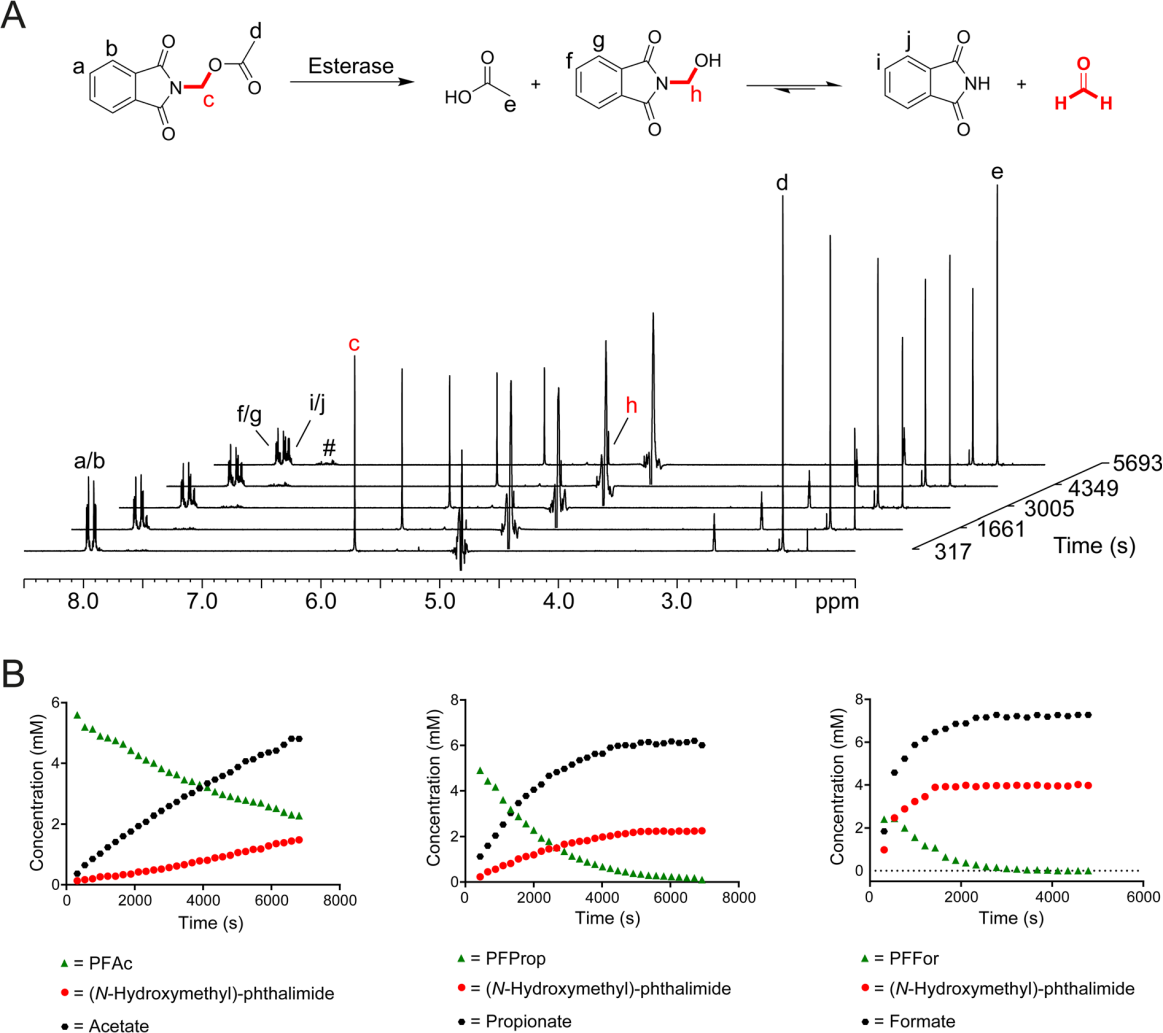
*N*-Acyloxymethyl-phthalimides release HCHO upon incubation with esterase. (A) ^1^H NMR spectra showing porcine esterase-catalysed fragmentation of PFAc. ^1^H resonances corresponding to phthalimide, acetate and (*N*-hydroxymethyl)-phthalimide are highlighted. (*N*-Hydroxymethyl)-phthalimide is a quasi-stable adduct of phthalimide and HCHO. Low-level formation of phthalamic acid derivatives was also observed (#). (B) Graphs showing time-dependent hydrolysis of PFAc (left), PFProp (middle), and PFFor (right) catalysed by porcine esterase.

To test whether release of HCHO from *N*-acyloxymethyl-phthalimides is induced by esterase catalysis, PFFor, PFAc, PFProp, PFPhen, PFBz, POxoBut and PGlycAc (100 μM in 100 mM phosphate buffer pH 7.5) were each incubated with isolated porcine esterase, a close homologue of human carboxylesterases (1 μM, total sample volume = 600 μL) at 25 °C, and the samples were monitored after 24 hours incubation by ^1^H NMR. For all compounds except POxoBut and PGlycAc, ^1^H resonances consistent with a mixture of both phthalimide and (*N*-hydroxymethyl)-phthalimide were observed after esterase treatment along with the appropriate carboxylate (Fig. 2A, S7 and S8).†^1^H resonances corresponding to additional species were also observed at low levels and increased in concentration over time. These resonances were similar to those observed for the phthalamic acid derivatives observed in DMEM with FBS. The characteristic ^1^H resonance of hydrated HCHO (*δ*_H_ 4.8 ppm) was not observed in the samples; however, this resonance is close to the residual water resonance and is therefore likely lost during water suppression. In the case of the control compound POxoBut, only ^1^H resonances corresponding to the starting material and a phthalamic acid derivative were observed, while ^1^H resonances corresponding to (*N*-hydroxyethyl)-phthalimide and acetate were detected in the sample with PGlycAc (note low-level formation of a phthalamic acid derivative was also observed at later time-points, potentially indicating hydrolysis of (*N*-hydroxyethyl)-phthalimide, Fig. S7†).

To determine whether esterase catalysis on the compounds released HCHO, dimedone (1 equivalent) was added to samples after incubation for 24 hours at 25 °C. ^1^H resonances corresponding to the hydroxymethyl-dimedone adduct were observed in all samples except for those containing POxoBut and PGlycAc (Fig. S8 and S9, Scheme S1†). Time-course experiments were then conducted. Samples were prepared containing one of the compounds (8.3 mM) and porcine esterase (1 μM, total sample volume = 600 μL) in the absence of dimedone and were monitored over 2 hours at 25 °C by ^1^H NMR. PFPhen and PFBz were only partially soluble in these samples; however, analysis of the intensities of ^1^H resonances corresponding to the carboxylate products enabled the determination of initial ester hydrolysis rates for PFAc, PFFor and PFProp (Fig. 2B). Of these compounds, PFAc was the least efficient substrate of porcine esterase (0.87 μM s^−1^), while PFProp and PFFor displayed faster hydrolysis rates (2.11 μM s^−1^ and 7.57 μM s^−1^ respectively). The faster rate of ester hydrolysis of PFFor is perhaps unsurprising given its observed hydrolysis in aqueous media (thus implying at least partial esterase-independent hydrolysis in the sample). However, it is also possible that PFFor is a more efficient substrate of porcine esterase than PFAc and PFProp, potentially due to steric factors (Fig. 2B and S10†). In all time-courses, evidence for (*N*-hydroxymethyl)-phthalimide was also apparent; however, conducting the experiment with PFAc in the presence of excess dimedone resulted in decreased levels of (*N*-hydroxymethyl)-phthalimide formation and induced formation of hydroxymethyl-dimedone (Fig. S10†). Therefore, it appears that (*N*-hydroxymethyl)-phthalimide is quasi-stable under the tested conditions.

Overall, these studies confirm that *N*-acyloxymethyl-phthalimides undergo esterase-catalysed hydrolysis of their ester bonds to release HCHO, phthalimide and a carboxylate. In addition, low-level non-enzymatic formation of *N*-acyloxymethyl-phthalamic acids occurs, although, esterase-catalysed ester hydrolysis is much more efficient under the tested conditions. It is also probable that *N*-acyloxymethyl-phthalamic acids are esterase substrates, leading to formation of phthalamic acid (or other derivatives) and HCHO release.

### *N*-Acyloxymethyl-phthalimides inhibit growth of human U2OS cells

**Fig. 3:**
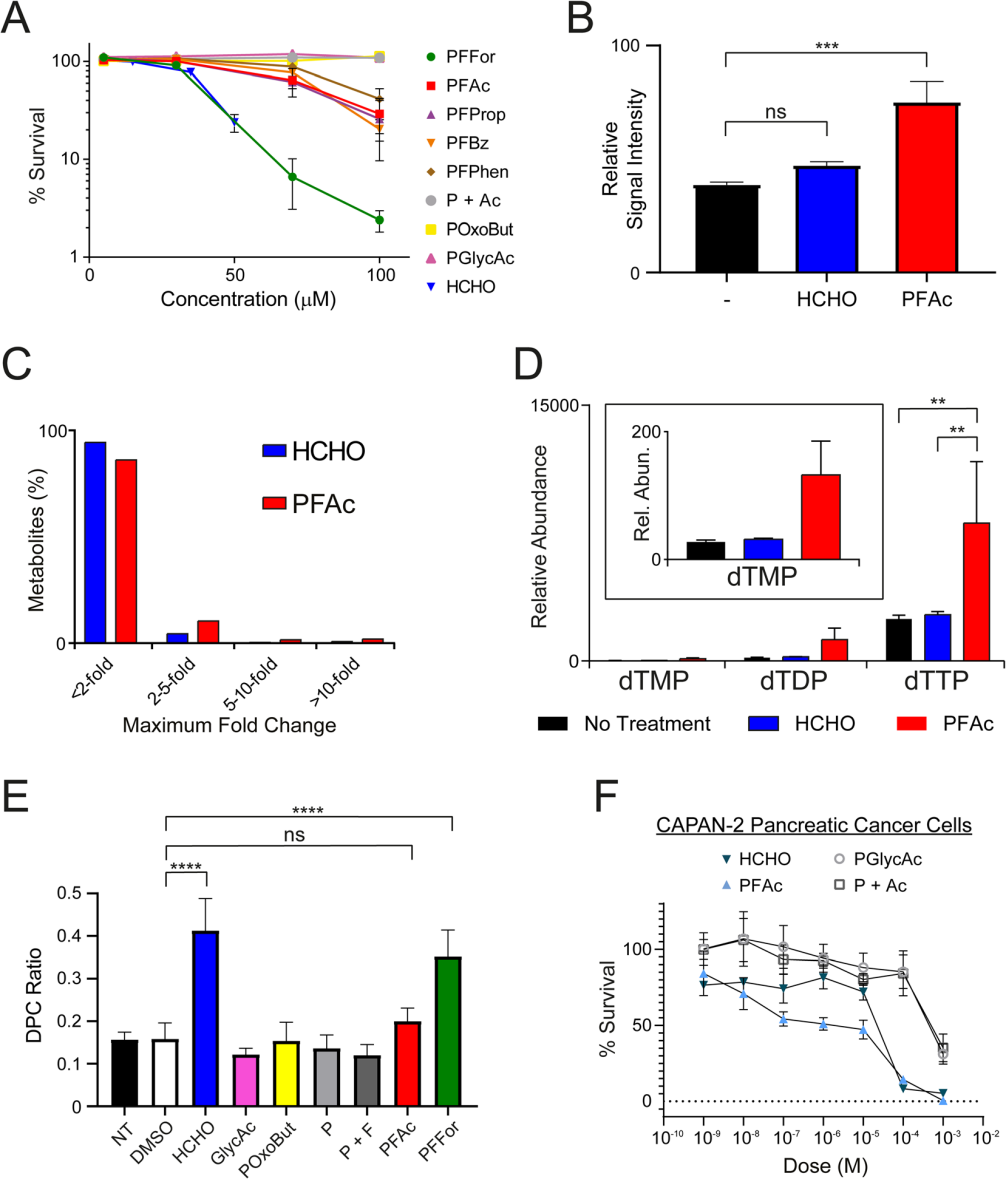
*N*-Acyloxymethyl-phthalimides deliver HCHO to cells. (A) PFFor, PFAc, PFProp, PFBz and PFPhen inhibit growth of human U2OS cells. Treatment with PFFor induces comparable growth inhibition to HCHO, while the other compounds are less inhibitory. Non-HCHO releasing analogue, PGlycAc, is not inhibitory over the tested concentration range. Combinatorial treatment with phthalimide and acetic acid (P + Ac) was also not inhibitory. The highest HCHO concentration tested was 50 μM. Incubations were for 12–16 days. Error bars represent standard errors of the mean (*n* = 4). (B) Cellular HCHO levels in U2OS cells increase after treatment with either HCHO or PFAc (100 μM). HCHO was quantified by monitoring formation of its hydroxymethyl adduct with dimedone, which was delivered at the point of cell lysis (1 mM). Error bars represent standard errors of the mean (*n* = 5). (C) Graph showing changes in abundance of identified metabolites in U2OS cells treated with either HCHO (blue) or PFAc (red). The majority of metabolites show little change in abundance (<2-fold) relative to untreated cells. (D) Increased levels of thymidine derivatives are observed in U2OS cells after treatment with HCHO (100 μM, blue) or PFAc (100 μM, red). Levels were increased more significantly in PFAc-treated cells. Error bars represent standard errors of the mean (*n* = 5). Note changes in dTMP and dTDP were not statistically significant (*P* values <0.05). (E) Treatment of U2OS cells with PFAc and PFFor (2 mM) leads to formation of DNA-protein cross-links (DPCs). NT = no treatment. Error bars represent standard deviations (*n* = 2–6). (F) MTT cytotoxicity assays showing concentration-dependent toxicity of HCHO, PFAc, PGlycAc and P + Ac in Capan-2 pancreatic cancer cells. HCHO and PFAc are more toxic than PGlycAc and P + Ac. Error bars represent standard deviations (*n* = 3). Annotation of *P* values is as follows; ns = ≥0.05, * = <0.05, ** = <0.01, *** = <0.001, **** = <0.0001.

Having determined that *N*-acyloxymethyl-phthalimides are HCHO-releasing compounds, we then conducted cellular studies. Initially, we assessed whether the compounds inhibit growth of human U2OS (osteosarcoma) cells. Seeded cells were treated with PFFor, PFAc, PFProp, PFPhen, PFBz, POxoBut, PGlycAc, or a combination of phthalimide and acetate (1 : 1 ratio, P + Ac), and were then incubated to allow growth of colonies (12–16 days at 37 °C). Colony counting revealed all HCHO-releasing compounds (PFFor, PFAc, PFProp, PFPhen and PFBz) inhibited growth at 100 μM (Fig. 3A and S11†). The formate- and HCHO-releasing compound PFFor was the most toxic, showing near-complete growth inhibition at 100 μM. With the exception of PFFor, however, all compounds were less inhibitory than HCHO. No growth inhibition was observed after treatment with POxoBut, PGlycAc, or P + Ac. While the increased inhibitory activity of PFFor relative to the other compounds might indicate more efficient HCHO release (as supported by the NMR studies), PFFor also releases formate, which can induce toxicity, *e.g.* by inhibiting mitochondrial cytochrome oxidase.^44^

### PFAc increases intracellular HCHO concentrations

We then attempted to observe HCHO release in U2OS cells after PFAc treatment. PFAc was selected as the lead compound for these and later experiments due to its good solubility in aqueous solution (up to 8.3 mM, comparable to that of PFFor and better than PFProp, PFPhen and PFBz) and for its resistance to HCHO-releasing degradation in media. It was also proposed that the acetate side-product of PFAc would induce little/no off-target bioactivity, thus simplifying the analyses. To quantify intracellular HCHO concentrations, U2OS cells were treated with either HCHO or PFAc (100 μM) for 2 hours at 37 °C before removal of the media, cell lysis and treatment with excess dimedone to scavenge the HCHO present. MS analysis of the resulting supernatant revealed evidence for the formation of the hydroxymethyl-dimedone adduct in all samples, suggesting U2OS cells have a detectable basal HCHO concentration (Fig. 3B). Nonetheless, treatment with either HCHO or PFAc resulted in an increase in hydroxymethyl-dimedone adduct levels, implying an elevation in cellular HCHO concentrations. Signal intensities for the hydroxymethyl-dimedone adduct were greatest upon PFAc treatment (1.9-fold increase relative to untreated control, *P* value = 0.001, Fig. 3B), which importantly suggests PFAc-mediated delivery of HCHO to cells is more efficient than treatment with free HCHO. This strongly implies that PFAc (and/or its phthalamic acid derivative) is entering cells before HCHO release.

### PFAc increases intracellular levels of thymidines

We next conducted metabolomics profiling of the treated samples using LC-MS. In total, 3080 individual compound features were detected across the samples, of which 101 were identified using authentic standards. While the majority of the compounds did not vary in concentration significantly across the samples, some marked differences in abundances were observed for a small number of metabolites (Fig. 3C). Notably, >3-fold increases in the concentrations of thymidine mono-, di- and triphosphates were observed in the samples with PFAc relative to those treated with either HCHO or untreated cells (Fig. 3D and Table S1,† note thymidine diphosphate is putatively assigned based on accurate mass only). While it is possible these changes are due to phthalimide or acetate, it is more likely a consequence of increased intracellular HCHO. HCHO is a key source of the methylene group in 5,10-methylenetetrahydrofolate, which is transferred to the 5-position of deoxyuridine monophosphate by thymidylate synthase, forming thymidine monophosphate.^8^ Given that the increase in thymidine biosynthesis appears more pronounced after PFAc treatment than after HCHO treatment, it is possible that PFAc induces greater localised HCHO concentrations that induces thymidine synthesis, *e.g.* co-localised with thymidylate synthase. Overall, the results suggest that HCHO levels regulate thymidine biosynthesis and imply that PFAc can be used as a tool to affect thymidine levels in cells.

### PFFor and PFAc induce DPCs

We then assessed whether *N*-acyloxymethyl-phthalimides can induce formation of DPCs. For these experiments, U2OS cells were either untreated, treated with DMSO as a carrier control, or treated with either HCHO, PGlycAc, POxoBut, phthalimide (P), a 1 : 1 ratio of phthalimide and formate (P + F, 2 mM each), PFAc or PFFor. The ratio of DNA bound to protein *versus* unbound DNA (DPC ratio) was used to measure the extent of DNA-protein crosslinking in each sample (Fig. 3E and Table S2†). No increase in DPC formation was observed with PGlycAc, POxoBut, P, or P + F; however, HCHO and PFFor induced statistically significant increases in DPC levels. HCHO induced slightly more DPCs by average than PFFor (2.61-fold and 2.22-fold increases respectively relative to the DMSO control), while the DPC ratio in the presence of PFAc was only marginally higher than control samples (1.26-fold relative to the DMSO control, not statistically significant on the basis of *P* values). The fewer DPCs observed with PFFor and PFAc relative to HCHO might suggest they induce lower effective HCHO concentrations in cell nuclei. This might be due to their localisation away from cell nuclei and/or their slow rate of HCHO release, thus leading to less acute but more prolonged exposure to HCHO. In this regard, it should be noted that the number of DPCs might not be linearly proportional to the nuclear HCHO concentration. For example, it is possible that base damage such as formation of apurinic/apyrimidinic (AP) damage sites induced by base excision repair of HCHO-derived adducts leads to formation of DPCs.^45^ Given that these processes require enzymes and that the fidelity of repair is dependent on multiple factors, it is likely that the time-scale for their formation is different than that for direct HCHO-induced cross-linking and might therefore be promoted under different conditions. These potential caveats further underscore the importance of developing tools that enable controlled delivery of HCHO in cells.

### PFAc induces death of pancreatic cancer cells

Finally, to establish effects on cancer cells of a different origin, we conducted cell growth inhibition (MTT) assays in two human pancreatic cancer cell lines. Here, we employed pancreatic cancer cell lines as these are generally resistant to most cytotoxic chemotherapeutics and represent a cancer of unmet clinical need. Indeed, sensitivity to HCHO might suggest new therapeutic strategies against pancreatic cancer. MTT assays were conducted on two cell lines, MIA-PaCa-2 and Capan-2 cells, which show differing sensitivities to the cancer drug gemcitabine, a frontline therapy in pancreatic cancer (MIA-PaCa-2: sensitive, Capan-2: resistant). Cultures were grown in 96 well plates for 24 hours before treatment with HCHO, PFAc, PGlycAc or P + Ac (1 nM to 1 mM) for 72 hours prior to the MTT assay. Interestingly, HCHO and PFAc were toxic to both MIA-PaCa-2 and Capan-2 cells but the effect was more pronounced in Capan-2 cells (Fig. 3F and S12†). HCHO and PFAc were comparably toxic in Capan-2 cells, inducing 92% and 86% cell death respectively at 100 μM (Fig. 3F). However, PGlycAc and P + Ac were only toxic to Capan-2 cells at 1 mM, which suggests that the toxicity of HCHO and PFAc is induced by elevated intracellular HCHO concentrations. No discernible differences in toxicity were observed for HCHO, PFAc, PGlycAc and P + Ac in MIA-PaCa-2 cells (Fig. S12†).

## Conclusions

Biological research on HCHO (and indeed many other reactive biomolecules) is hindered by a lack of validated, easily prepared and bench-stable delivery agents. To address this limitation, we here report a set of *N*-acyloxymethyl-phthalimides as cell-relevant HCHO-releasing compounds. Our studies indicate that *N*-acyloxymethyl-phthalimides are bench-stable solids that (i) are stable in DMSO stock solutions and can be highly soluble in aqueous buffer and cell media (up to 8.3 mM in the case of lead compound PFAc), (ii) are easily prepared in one step from readily available starting materials, (iii) are stable to HCHO release in cell media, and (iv) controllably release HCHO in cells as a consequence of enzymatic ester hydrolysis. Our cellular experiments with lead compound PFAc and control compounds suggest *N*-acyloxymethyl-phthalimides can increase intracellular HCHO concentrations, can affect cancer cell metabolism, can induce DPCs, and can kill gemcitabine-resistant pancreatic cancer cells. These results suggest *N*-acyloxymethyl-phthalimides mimic endogenous HCHO production and therefore offer an attractive alternative to HCHO in cellular assays. The results also suggest that *N*-acyloxymethyl-phthalimides, and by extension other HCHO-releasing compounds, can inhibit the growth and/or induce the death of human cancer cells. This finding suggests HCHO releasers have potential in cancer chemotherapy.

In conclusion, we propose that our *N*-acyloxymethyl-phthalimides are more quantitative, controllable and reproducible alternatives to free HCHO and genetic methods for modulating HCHO concentrations in cells. Collectively, their relatively simple synthesis, their stability and easy handling as solids and as DMSO stock solutions, their stability in media, their susceptibility to esterases, and their relative lack of toxicity compared to free HCHO, imply they can be used in a wide variety of cellular assays as HCHO donors (note: it is likely that many hydrolytic enzymes in cells will induce HCHO release, ensuring their broad applicability). The fact that our *N*-acyloxymethyl-phthalimides release bio-orthogonal phthalimide and carboxylates alongside HCHO (not the case for acyloxymethyl group-containing pro-drugs), as well as our use of non-HCHO-releasing control compounds, also suggest that any phenotypic changes observed in cellular experiments can be attributed to HCHO release. Given the application of acyloxymethyl groups in pro-drugs, our results also emphasise the potential for such compounds to affect human cell biology *via* HCHO release. This may lead to off-target toxicity, although there is also the potential for advantageous dual-action therapies in cancer patients.

## Data availability

All data are available from the corresponding authors on reasonable request.

## Author contributions

L. P. S, P. J. M. and R. J. H. conceived the study. F. W. M., L. A. L., L. B., N. F. A. B., R. J. H. and V. L. E. conducted the synthetic and compound characterisation experiments, L. A. L. conducted the NMR studies with esterase, L. P. S., R. J. H. and V. L. E. conducted the cellular experiments, and E. P. and J. S. O. M. conducted the cellular mass spectrometry analyses. E. P., L. A. L., L. P. S., P. J. M. R. J. H. and V. L. E. wrote the manuscript. All authors commented on the manuscript.

## Conflicts of interest

There are no conflicts to declare.

## Supplementary Material

SC-014-D3SC02867D-s001
