# Supplementary material for: N-Acyloxymethyl-phthalimides deliver genotoxic formaldehyde to human cells
Source: Chem Sci. 2023 Sep 15;14(44):12498–505. doi: 10.1039/d3sc02867d (PMC10646869; doi:10.1039/d3sc02867d)
Supplement: SC-014-D3SC02867D-s001 [file SC-014-D3SC02867D-s001.pdf]

Electronic Supplementary Material (ESI) for Chemical Science.
This journal is © The Royal Society of Chemistry 2023

Supplementary Information

N-acyloxymethyl-phthalimides deliver genotoxic formaldehyde to human cells

Vicki L. Emms,[†a] Liam A. Lewis,[†a] Lilla Beja,[a] Natasha F. A. Bulman,[a] Elisabete Pires,[b] Frederick W. Muskett,[c] James S. O. McCullagh,[b] Lonnie. P. Swift,[*d] Peter J. McHugh,[*d] and Richard J. Hopkinson[*a]

[a]Leicester Institute for Structural and Chemical Biology and School of Chemistry, University of Leicester, Henry Wellcome Building, Lancaster Road, Leicester, LE1 7RH, UK. E-mail: richard.hopkinson@leicester.ac.uk

[b]Department of Chemistry, University of Oxford, Chemistry Research Laboratory, 12 Mansfield Road, Oxford, OX1 3TA, UK.

[c]Leicester Institute for Structural and Chemical Biology and Department of Molecular and Cell Biology, University of Leicester, Henry Wellcome Building, Lancaster Road, Leicester, LE1 7RH, UK.

[d]Department of Oncology, University of Oxford, MRC Weatherall Institute of Molecular Medicine, University of Oxford, John Radcliffe Hospital, Headington, Oxford, OX3 9DS, UK, E-mail: lonnie.swift@imm.ox.ac.uk, peter.mchugh@imm.ox.ac.uk

[†]The authors wish it to be known that, in their opinion, the first two authors should be regarded as Joint First Authors

<u>Materials and Methods</u>

Synthesis

N-acyloxymethyl-phthalimides were synthesised using the following procedure. Carboxylic acid (1.1 mmol) was added to a solution of (*N*-bromomethyl)-phthalimide (240 mg, 1 mmol) in EtOAc (5 mL) in a glass vial. DIPEA (192 µL, 1.1 mmol) was then added dropwise and the mixture was stirred at room temperature (8-12 hours). CH_2Cl_2 (10 mL) was then added and the mixture was transferred to a round-bottomed flask. Removal of the solvent *in vacuo* gave a white solid, which was redissolved in CH_2Cl_2 and purified by flash chromatography on silica gel (conditions: 95 % CH_2Cl_2, 5 % MeOH, 10 CV). The product was isolated after evaporation of the solvent *in vacuo*.

For spectroscopic characterisation of all compounds, as well as the procedures for the syntheses of PGlycAc and POxoBut, please see below. The purity of all compounds was ≥95 %.

Stability Studies

N-acyloxymethyl-phthalimide or control compound (50 µL of a 100 mM stock in DMSO), TSP (10 µL of a 10 mg/mL solution in H_2O), D_2O (65 µL) and Dulbecco Modified Eagle Medium media with 10 % v/v heat-inactivated foetal bovine serum (DMEM with FBS, 600 µL) was mixed in a microcentrifuge tube, transferred to a 5 mm diameter NMR tube and incubated at 37 °C for 24 hours. Due to the poor solubility of PFProp, 575 µL of DMEM with FBS containing 25 µL of DMSO was used. Degradation of the compounds was observed by ^{1}H NMR spectroscopy with water suppression (pre-saturation) using a Bruker AV500 NMR spectrometer equipped with a BBO probe and installed with TopSpin 4.1 software. After 24 hours, dimedone (7.5 µL of a 1 M stock in H_2O), was added to each mixture and the samples were re-analysed by ^{1}H NMR spectroscopy. Characterisation of the PFAc degradation product were conducted on a sample containing PFAc (50 µL of a 100 mM stock in DMSO-D_6), TSP (10 µL of a 10 mg/mL stock in D_2O), D_2O (150 µL), and 100 mM sodium phosphate buffer in D_2O pH 7.5 (390 µL). After mixing in a microcentrifuge tube, the sample was transferred to a 5 mm NMR tube and incubated at 37 °C for 24 hours. ^{1}H-^{13}C-HSQC and ^{1}H-^{13}C-HMBC

analyses were then conducted on a Bruker Avance NEO 800 MHz spectrometer equipped with a TCI cryoprobe.

NMR analyses with porcine esterase

Samples for NMR analyses (600 μL total volume) contained porcine esterase (10 μL of a 60 mM stock in 100 mM phosphate buffer in H_2O pH 7.5), an *N*-acyloxymethyl-phthalimide or control compound (0.6 μL or 50 μL of a 100 mM stock in DMSO), DMSO (0 μL – 30 μL), either H_2O (0.6 μL) or dimedone (0.6 μL of a 100 mM stock in H_2O), D_2O (150 μL), and 100 mM phosphate buffer in H_2O pH 7.5 (355 – 434 μL). After mixing in microcentrifuge tubes, the samples were transferred to 5 mm diameter NMR tubes and monitored by ^1H NMR with water suppression (pre-saturation) using a Bruker AV500 NMR spectrometer equipped with a 5 mm BBO probe and installed with TopSpin 3.6.1 software. Each ^1H NMR experiment consisted of 16 transients and 224 seconds of acquisition time. For time-course studies, each analysis consisted of 29 ^1H NMR experiments running consecutively, giving a total acquisition time of 109 – 120 minutes. Reaction rates were determined by measuring the intensities of the ^1H resonances corresponding to the carboxylate products over time and comparing them to the intensity of the ^1H resonance of the TSP standard. Rates are reported in μM s^{-1}.

Growth inhibition assays

Human U2OS (osteosarcoma) cells were seeded in 10 cm diameter petri dishes and incubated in 10 mL of DMEM with FBS for 24 hours at 37 °C. The dishes were then treated (10 μL) with either DMSO, HCHO in DMSO, an *N*-acyloxymethyl-phthalimide in DMSO or control compound in DMSO (5-100 μM final concentration). The dishes were then incubated for 12-16 days at 37 °C (5 % CO_2) to allow growth of colonies. The dishes were then drained of media and washed with phosphate-buffered saline (PBS). Coomassie Blue stain (0.1 % Coomassie Brilliant Blue R-250, 7 % glacial acetic acid, 50 % methanol in H_2O) was added to stain colonies before draining and washing with H_2O. Colonies were counted using an Oxford Optronix Colcount cell counter.

Mass spectrometry analyses

U2OS cells were grown to confluence in 25 cm^2 flasks at 37 °C containing Dulbecco Modified Eagle Medium containing 10 % v/v heat-inactivated foetal bovine serum before addition of HCHO or PFAc (100 μM final concentration). After two hours incubation at 37 °C, the media was removed and the cells were washed with PBS. A 4:1 CH_3CN:H_2O mix containing 1 mM dimedone (500 μL) was then added to each flask to induce cell lysis and scavenge HCHO. The mix was pipetted into a microcentrifuge tube and centrifuged at 13000 rpm for 30 minutes. The supernatant was then removed and filtered through a 10 kDa cut-off syringe filter. Samples were stored at -80 °C until the day of analysis. Each sample was thawed and then analysed by ion-exchange chromatography coupled directly to high-resolution mass spectrometry using the method previously described[1]. P values (here and elsewhere) were determined by the ANOVA significance test using GraphPad Prism 9.3.1.

Detection of DPCs

DPCs were measured as previously described[2]. Briefly, 5 x 10^5 U2OS cells either untreated or treated for 2 hours with either DMSO (carrier control), HCHO, an *N*-acyloxymethyl-phthalimide, or control compound, and were then washed twice with DMEM with FBS and lysed by scraping in 400 μL of denaturing lysis buffer (2 % SDS, 20 mM Tris.HCl pH 7.5). The cell lysate was then frozen at -80 ºC. Sample processing involved thawing at 55 ºC for 5 minutes and sonication at maximum attenuation for three cycles of 20 seconds on, 20 seconds off (on ice). This lysate was then pelleted at 15000 x g. The supernatant was then removed and saved as the soluble DNA fraction. The pellet fraction was washed three times by being resuspended in 400 μL of TK buffer (20 mM Tris pH 7.5, 200 mM KCl), shaken at 55 ºC for 5 minutes, cooled on ice for 5 minutes and then pelleted at 15000 x g for 5 minutes. The samples

were resuspended in TK buffer and proteins digested with 0.2 mg/mL Proteinase K at 55 ºC for 45 minutes. Bovine serum albumin (BSA, 500 µg) was added to each sample, which was then cooled on ice for 5 minutes before undergoing final centrifugation. The supernatant containing the initially cross-linked DNA was then treated with RNAse A (0.2 mg/mL) for 30 minutes at 37 ºC. DNA concentrations of the soluble fraction and the cross-linked fraction (20 µL each) were determined using the Qubit dsDNA HS assay according to the manufacturer's instructions (ThermoFisher Scientific) and presented as a ratio. With the exception of the experiment with P + F, all experiments were conducted at least twice on different occasions. Averaged values and standard deviations are calculated from all replicates (n = 2-6).

Cytotoxicity assays

Cell viability was determined by a colorimetric MTT assay. Cell suspension (100 µL, 1 x 10^5 cells/mL) was added each well of a 96-well plate and incubated overnight at 37 °C, 5 % CO_2 to allow cells to adhere. Media was aspirated and cells were treated with varying concentrations of HCHO or phthalimide-containing compound in fresh media (200 µL with 1 % DMSO), and then incubated for 72 hours at 37 °C, 5 % CO_2. After treatment, 20 µL of MTT solution (5 mg/mL in PBS) was added to each well and incubated for a further 4 hours. The media was then aspirated and the resultant formazan crystals were dissolved in DMSO (50 µL). The absorbance intensity at 570 nm was then measured using a Hidex Sense microplate reader. All experiments were performed in triplicate and the relative cell viability (%) was expressed as a percentage relative to the untreated control cells. No colour change was observed in control experiments containing MTT and either HCHO or PFAc (no cells, 4 hours incubation at 37 °C).

Supplementary Figures and Schemes

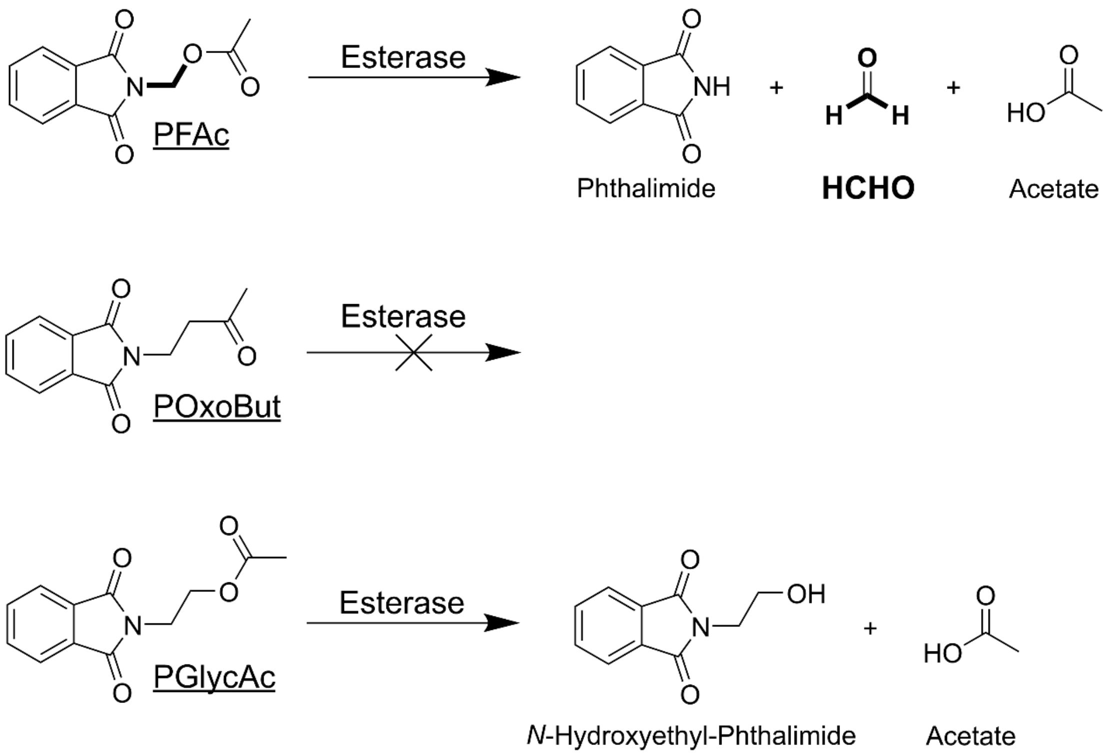

Scheme S1. Proposed products of esterase catalysis on *N*-acyloxymethyl-phthalimides and control compounds. (Top) scheme showing esterase-catalysed hydrolysis of *N*-acyloxymethyl-phthalimides. Hydrolysis produces phthalimide, acetate and HCHO. The PFAc analogue POxoBut is not an esterase substrate (middle); PGlycAc is hydrolysed by esterase to release acetate and N-hydroxyethyl-phthalimide, but not HCHO (bottom).

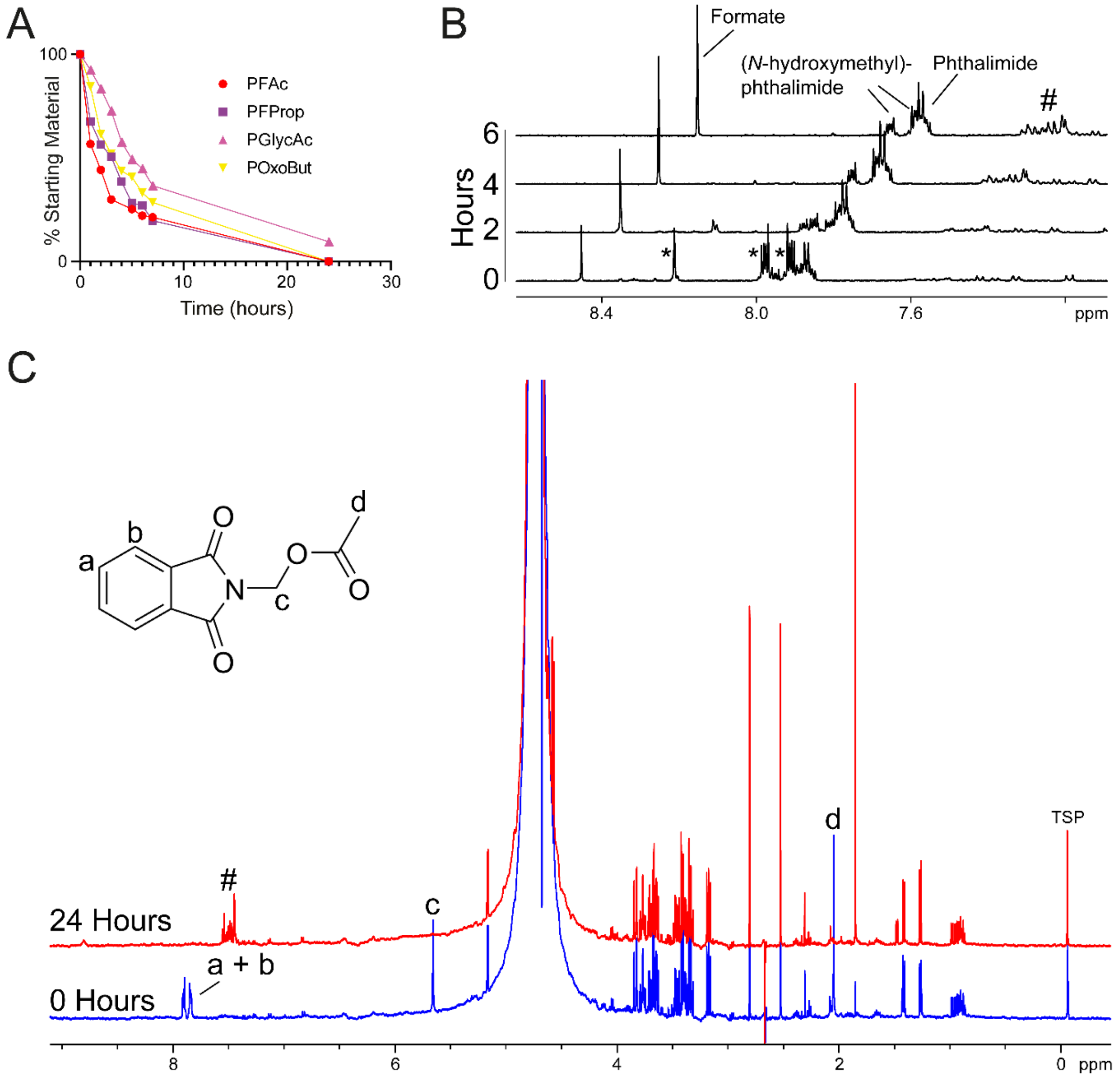

Figure S1. *N*-acyloxymethyl-phthalimides undergo degradation in cell media. (A) Graph showing time-dependent degradation of PFAc, PFProp, PGlycAc and POxoBut in Dulbecco Modified Eagle Medium containing 10 % v/v heat-inactivated foetal bovine serum (DMEM with FBS) at 37 °C. (B) Offset ^{1}H NMR spectra showing time-dependent degradation of PFFor in DMEM with FBS at 37 °C. Formation of formate, phthalimide and (*N*-hydroxymethyl)-phthalimide is observed. ^{1}H resonances assigned to phthalamic acid species are highlighted (hash). ^{1}H resonances corresponding to PFFor are marked by asterisks. (C) ^{1}H NMR spectra of a sample containing PFAc in DMEM with FBS incubated at 37 °C for 0 and 24 hours. ^{1}H resonances assigned to phthalamic acid species are highlighted (hash). ^{1}H resonances corresponding to PFAc are labelled a-d. TSP = 3-(trimethylsilyl)propionic-2,2,3,3-d_4 acid.

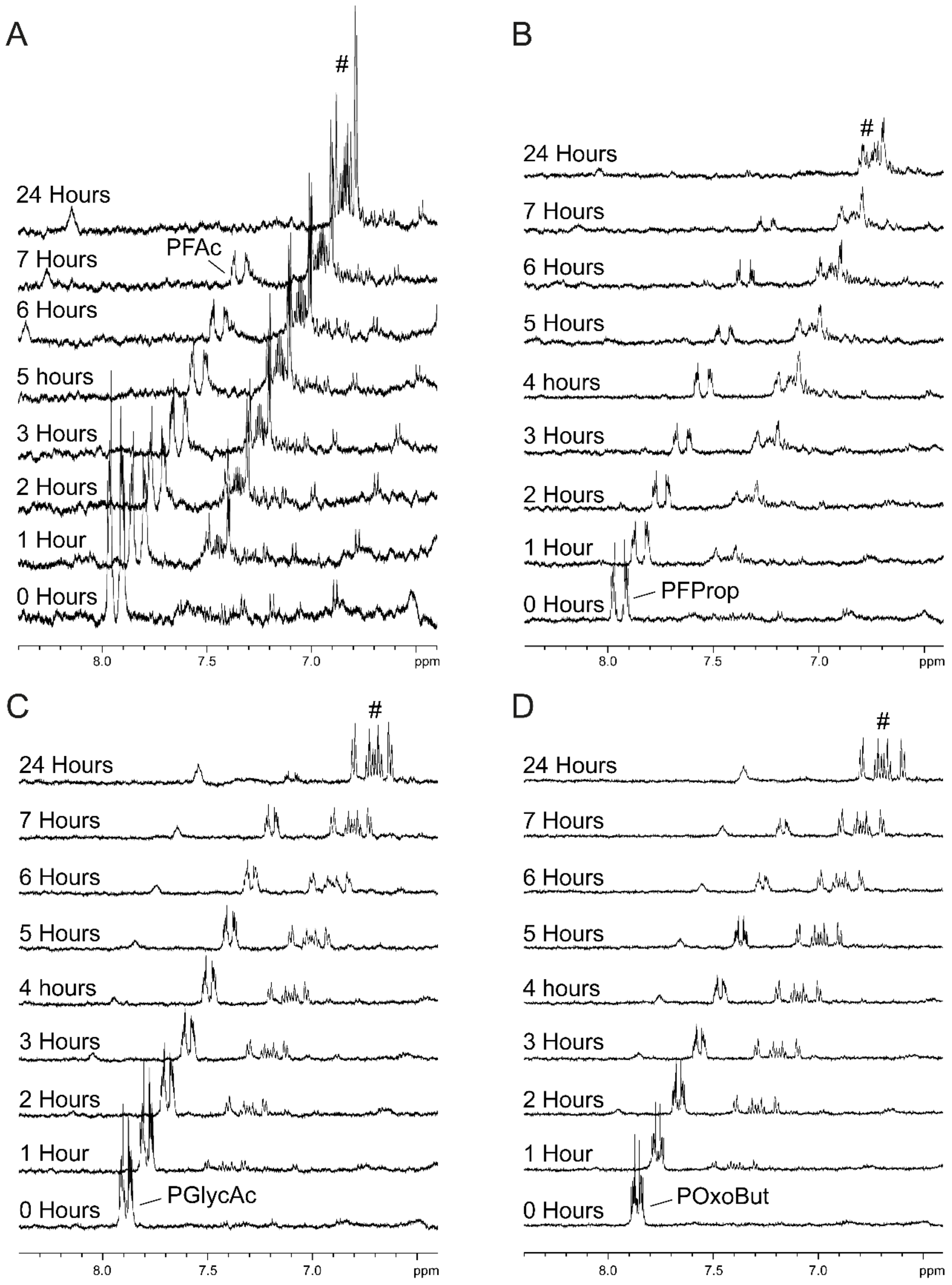

Figure S2. Offset ^{1}H NMR spectra showing time-dependent degradation of PFAc (A), PFProp (B), PGlycAc (C), and POxoBut (D) in DMEM with FBS at 37 °C. No significant formation of phthalimide or (*N*-hydroxymethyl)-phthalimide is observed. ^{1}H resonances assigned to phthalamic acid species are highlighted (hash).

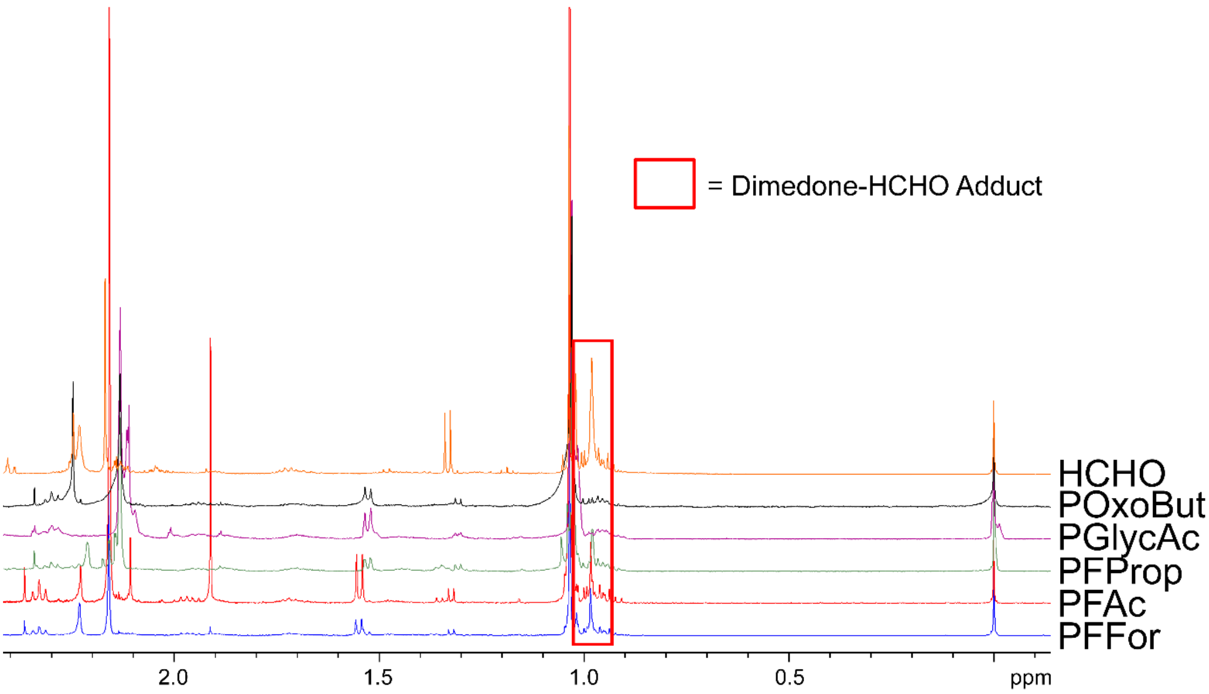

Figure S3. ^{1}H NMR spectra of samples containing PFFor, PFAc, PFProp, PGlycAc, POxoBut and HCHO in DMEM with FBS after addition of dimedone. ^{1}H resonances corresponding to the dimedone-HCHO adduct are highlighted. Note the sample with PFFor was incubated for 27 hours before addition of dimedone, whereas the samples with PFAc, PFProp, PGlycAc, POxoBut and HCHO were incubated for 24 hours. Note HCHO scavenging by dimedone is unlikely to be quantitative in these complex mixtures due to competing reactions with residual phthalimide (as is present in the sample with PFFor) and media components. The sample with HCHO was with a different DMEM with FBS batch of marginally higher concentration.

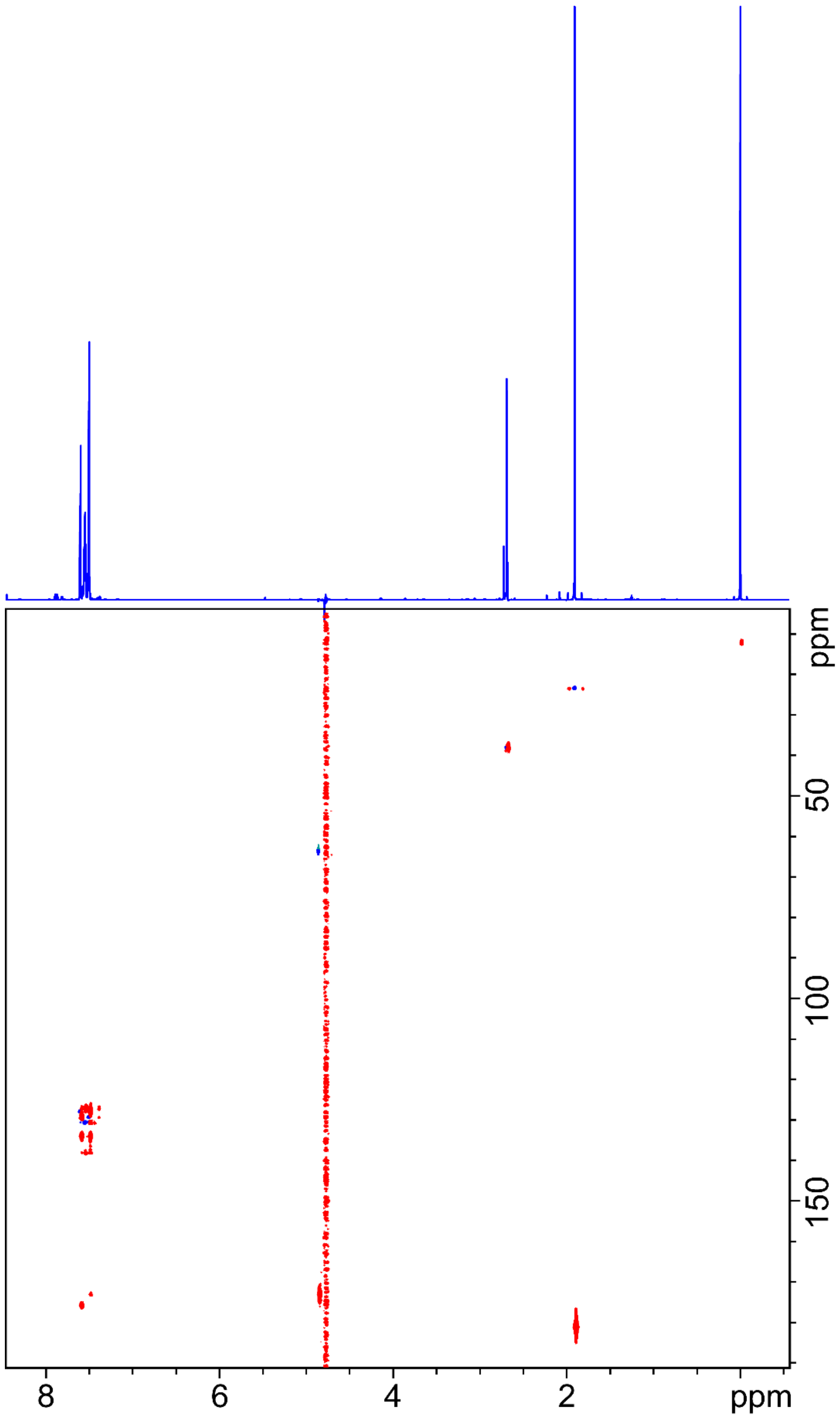

Figure S4. Overlay of 2D ^1H-^{13}C-HSQC (blue/green) and ^1H-^{13}C-HMBC (red) spectra of a sample of PFAc in D_2O pre-incubated for 24 hours at 37 °C.

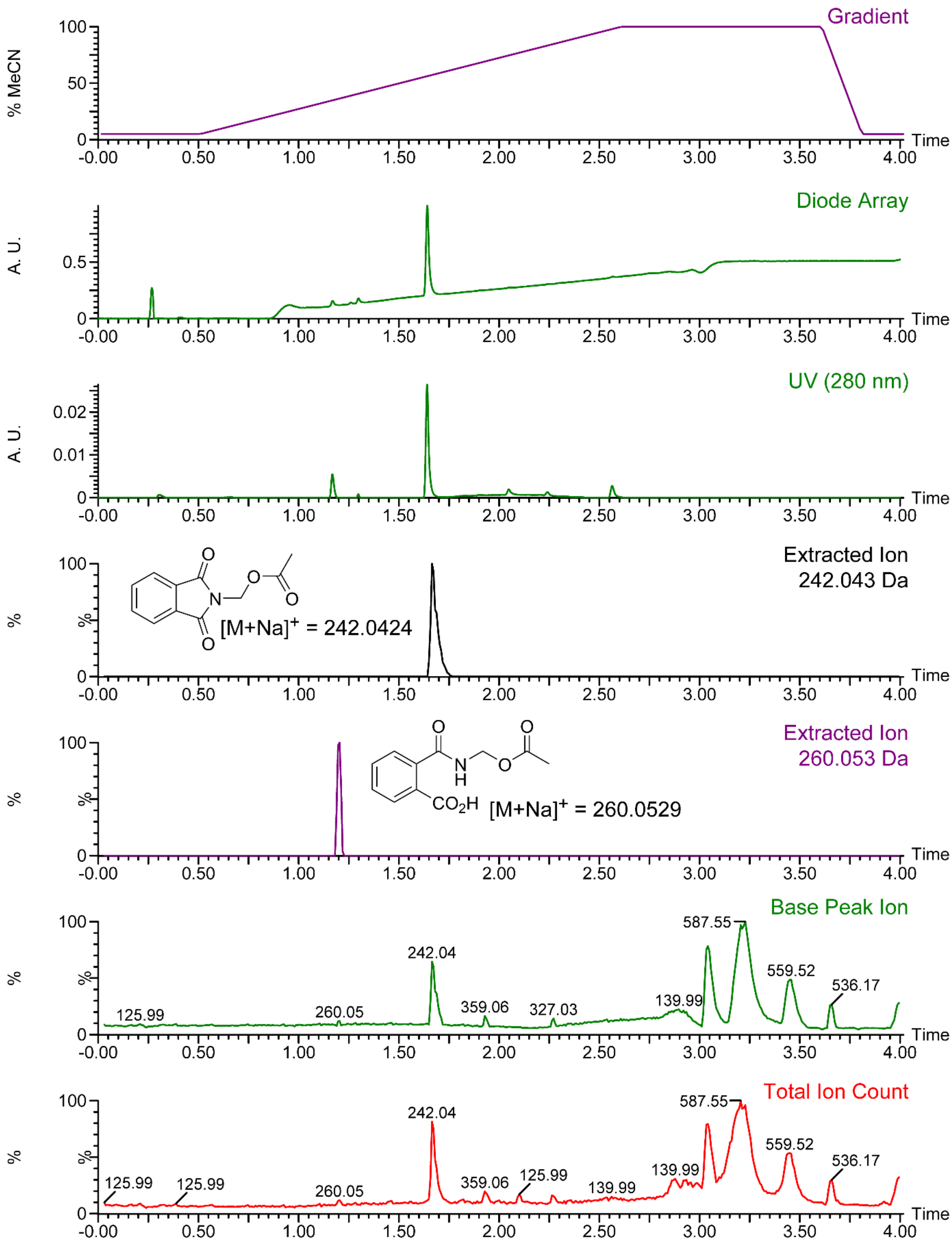

Figure S5. LC/MS analysis of a sample of PFAc in 1:1 water:acetonitrile after 24 hours. The presence of both PFAc and its *N*-acyloxymethyl-phthalamic acid derivative is evidenced by peaks after 1.64 minutes and 1.17 minutes respectively. Note degradation of PFAc is less proficient in water/acetonitrile than in water, 100 mM phosphate buffer pH 7.5, or DMEM with FBS.

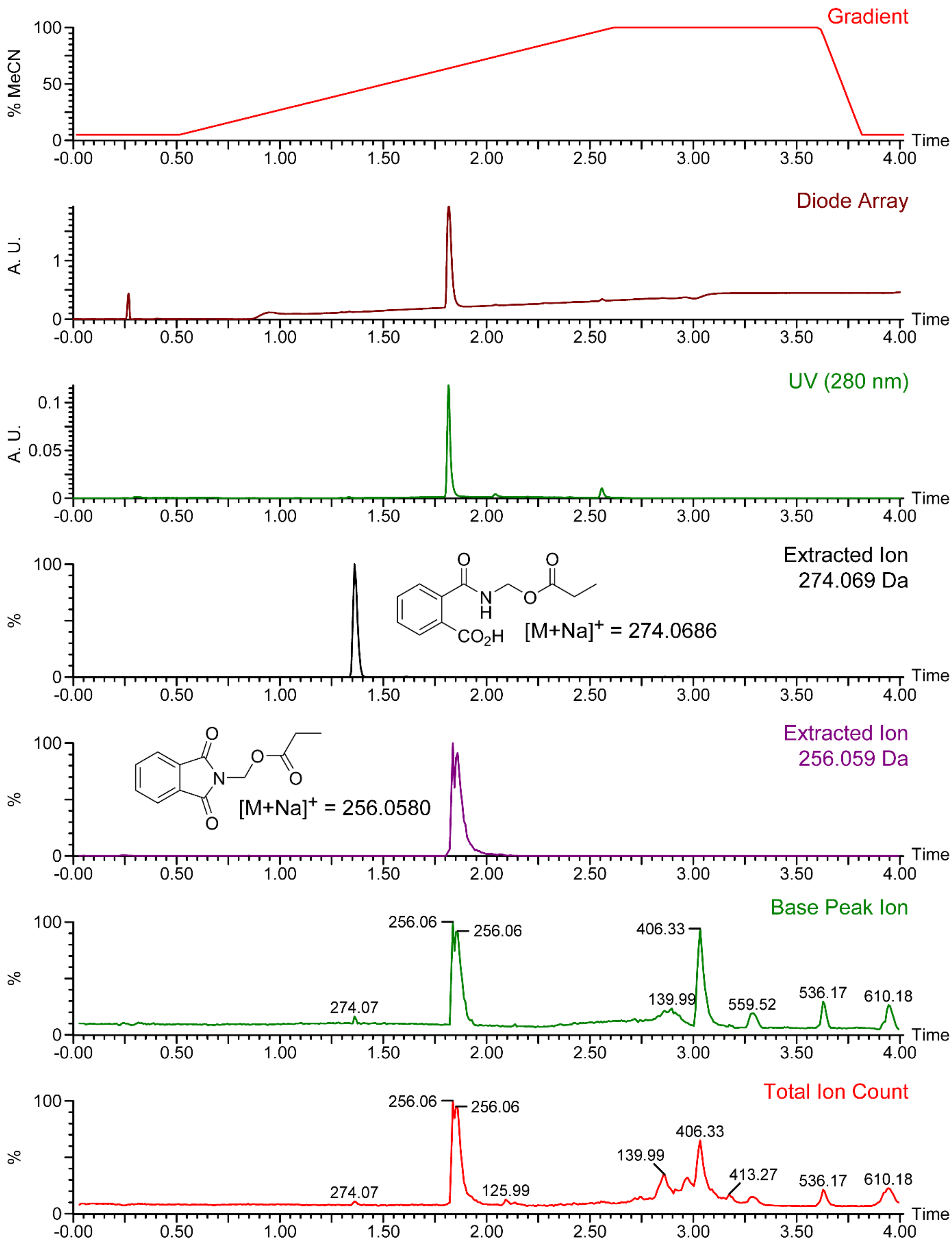

Figure S6. LC/MS analysis of a sample of PFProp in 1:1 water:acetonitrile after 24 hours. The presence of both PFProp and its *N*-acyloxymethyl-phthalamic acid derivative is evidenced by peaks after 1.82 minutes and 1.36 minutes respectively. Note degradation of PFProp is less proficient in water/acetonitrile than in water, 100 mM phosphate buffer pH 7.5, or DMEM with FBS.

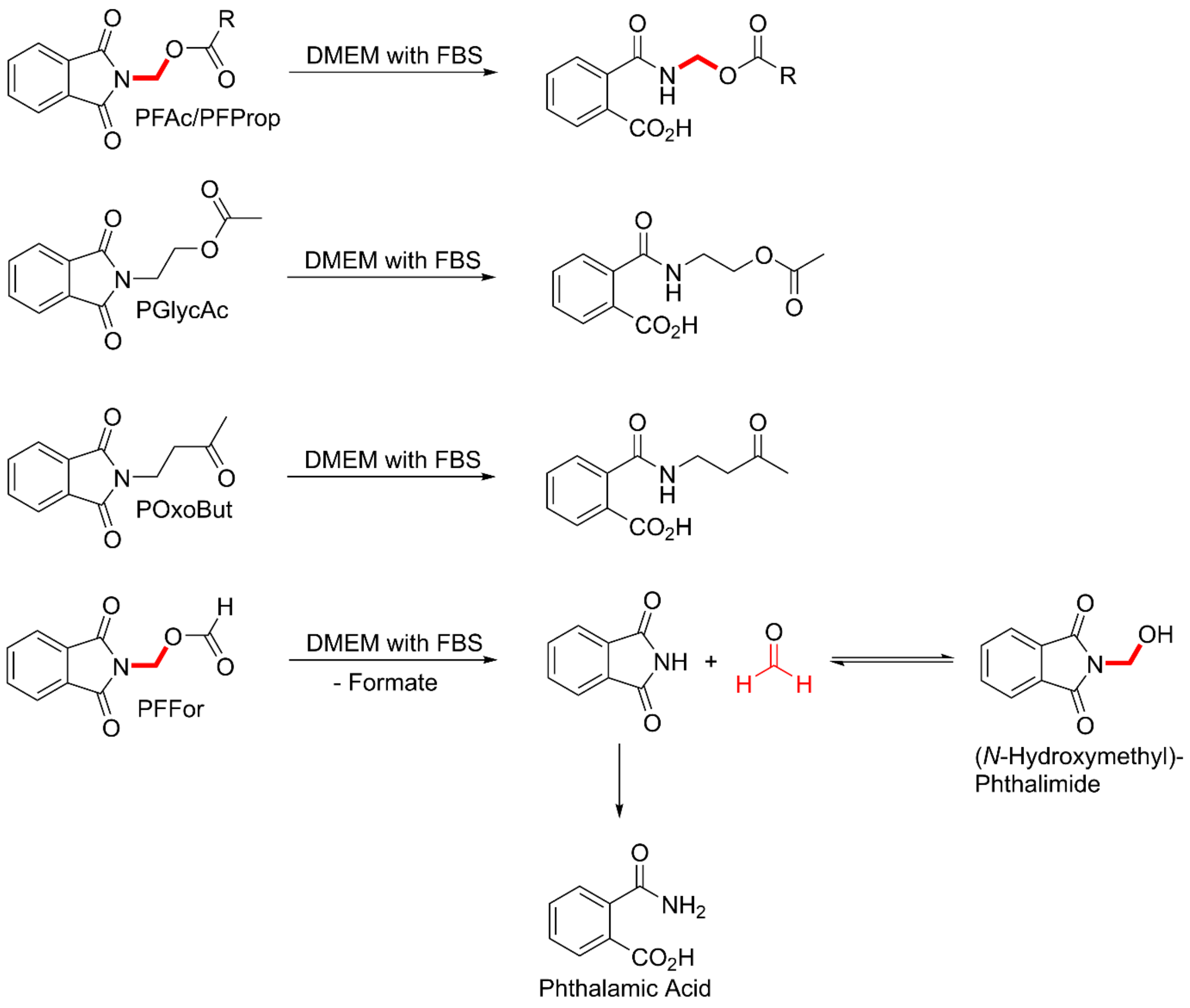

Scheme S2. Proposed major degradation products of *N*-acyloxymethyl-phthalimides and control compounds incubated in DMEM with FBS at 37 °C. PFFor appears to undergo HCHO-releasing ester hydrolysis (bottom), whereas PFAc and PFProp undergo imide hydrolysis to give *N*-acyloxymethyl-phthalamic acids (top). Similar phthalamic acid species are observed with the control compounds PGlycAc and POxoBut (middle). Note formation of other presumably low-level phthalamic acid species may occur but signal overlap precluded their detection/characterisation.

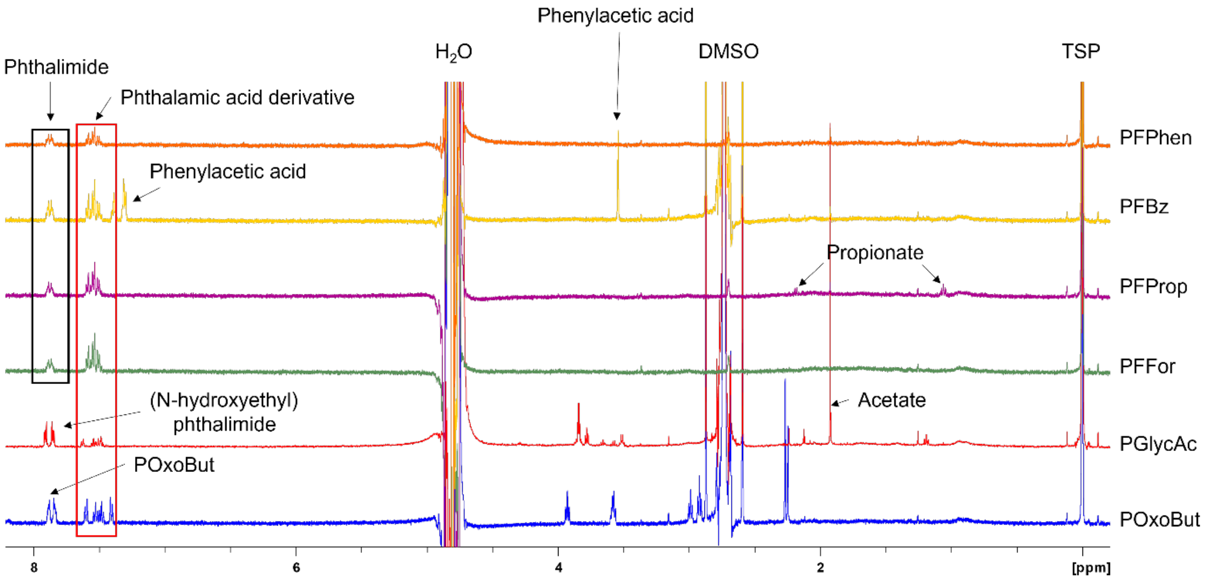

Figure S7. ^{1}H NMR spectra of samples containing porcine esterase (1 µM) and either PFPhen (orange), PFBz (yellow), PFProp (purple), PFFor (green), PGlycAc (red) or POxoBut (blue, all at 100 µM) in 100 mM phosphate buffer pH 7.5 after 24 hours incubation at 25 °C. ^{1}H resonances corresponding to the phthalimide product (black box) and carboxylate products are highlighted. Note ^{1}H resonances for benzoic acid (after hydrolysis PFPhen) are obscured by signal overlap. Degradation to phthalamic acid derivatives is also observed (red box). PGlycAc undergoes hydrolysis to (*N*-hydroxyethyl)-phthalimide and acetate (with some phthalamic acid-type product(s)), while POxoBut is susceptible to partial degradation to a phthalamic acid derivative.

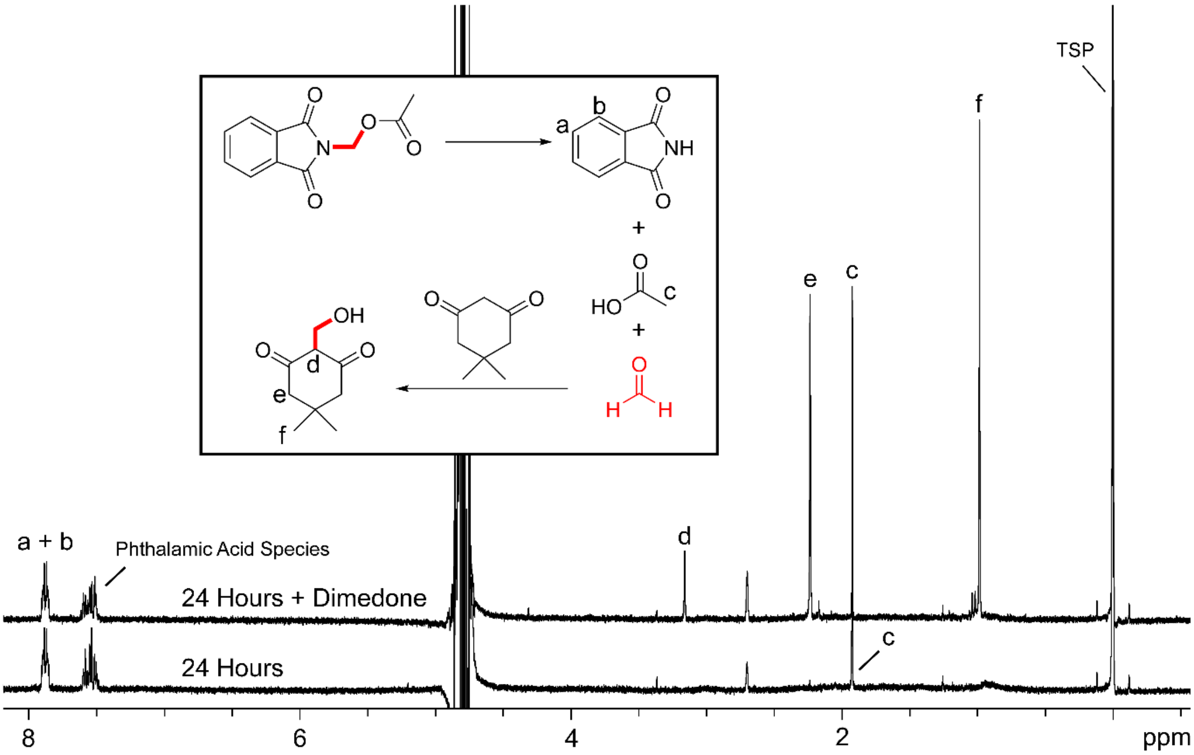

Figure S8. ^{1}H NMR spectra showing porcine esterase-catalysed fragmentation of PFAc (24 hours incubation at 25 °C) in the absence (bottom) and presence (top) of the HCHO scavenger dimedone. ^{1}H resonances corresponding to phthalimide, acetate and the dimedone-HCHO adduct hydroxymethyl-dimedone are highlighted.

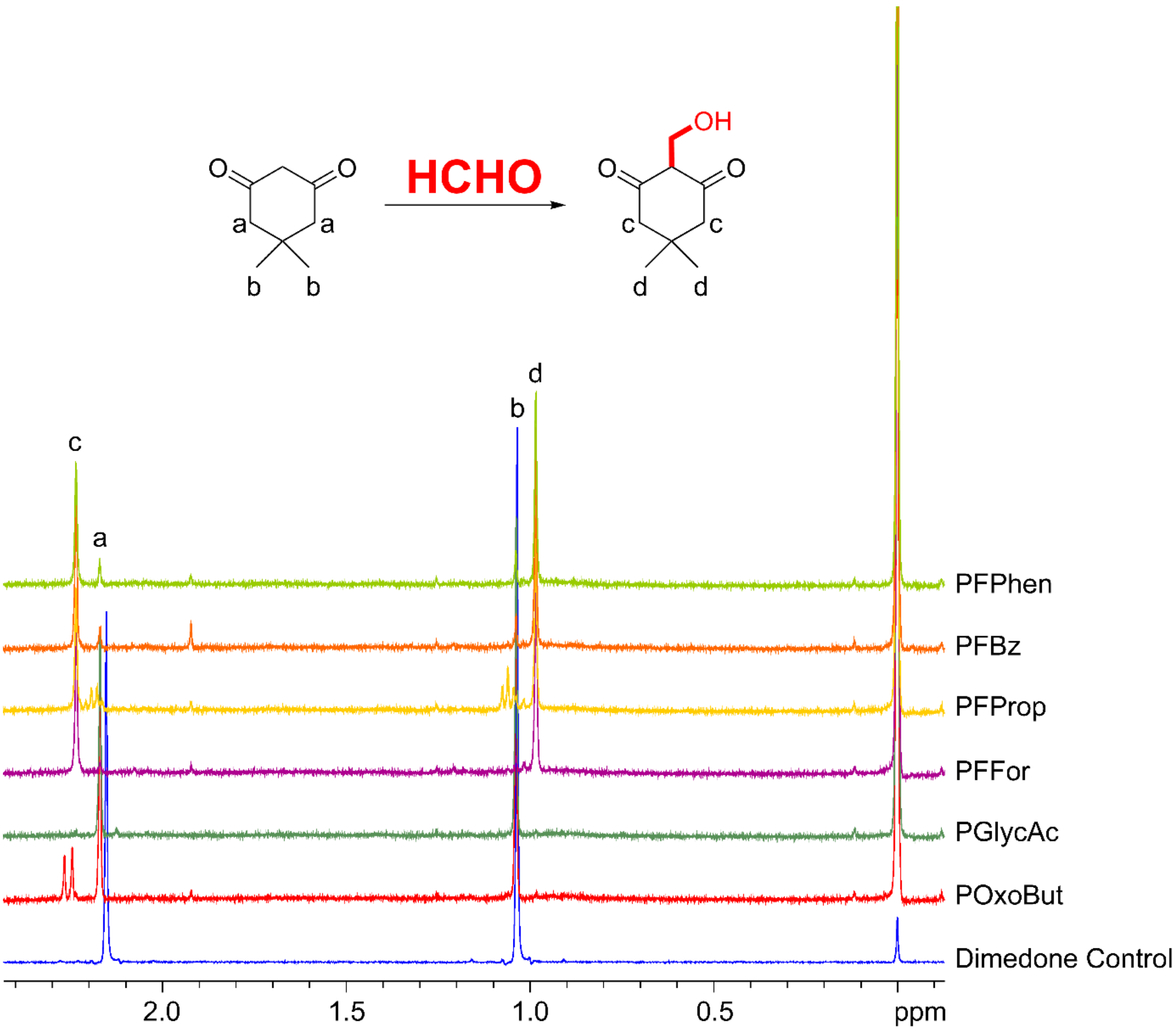

Figure S9. ^{1}H NMR spectra of samples containing porcine esterase (1 μM) and either PFPhen (light green), PFBz (orange), PFProp (yellow), PFFor (purple), PGlycAc (green) or POxoBut (red, all at 100 μM) in 100 mM phosphate buffer pH 7.5 after 24 hours incubation at 25 °C before addition of dimedone (0.6 μL of a 100 mM stock in H_2O). ^{1}H resonances corresponding to the dimedone-HCHO adduct are highlighted. A control sample containing dimedone is shown in blue. The ^{1}H resonance at δ_H 0 ppm corresponds to TSP.

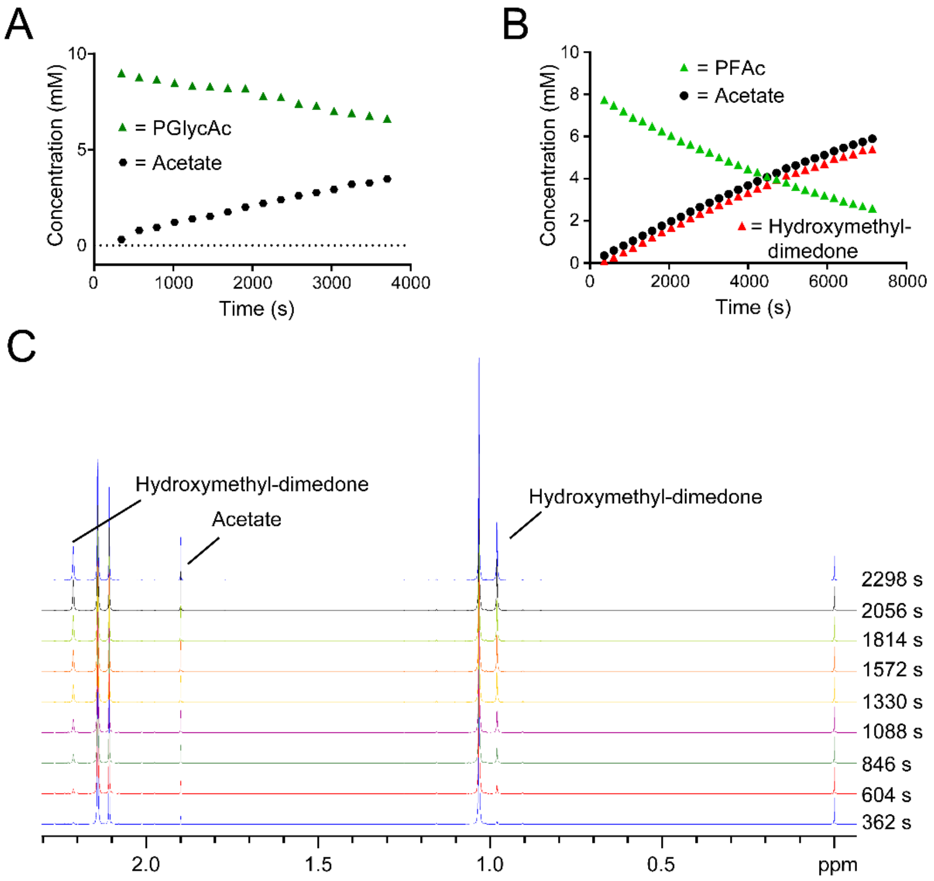

Figure S10. (A) Graph showing time-dependent hydrolysis of PGlycAc catalysed by porcine esterase. (*N*-hydroxyethyl)-phthalimide and acetate are produced during catalysis. (B) Graph showing time-dependent hydrolysis of PFAc catalysed by porcine esterase in the presence of excess dimedone. Formation of acetate and hydroxymethyl-dimedone are highlighted. (*N*-hydroxymethyl)-phthalimide was only observed at low levels. (C) ^{1}H NMR spectra showing time-dependent hydrolysis of PFAc catalysed by porcine esterase in the presence of excess dimedone. ^{1}H resonances corresponding to acetate and hydroxymethyl-dimedone are highlighted.

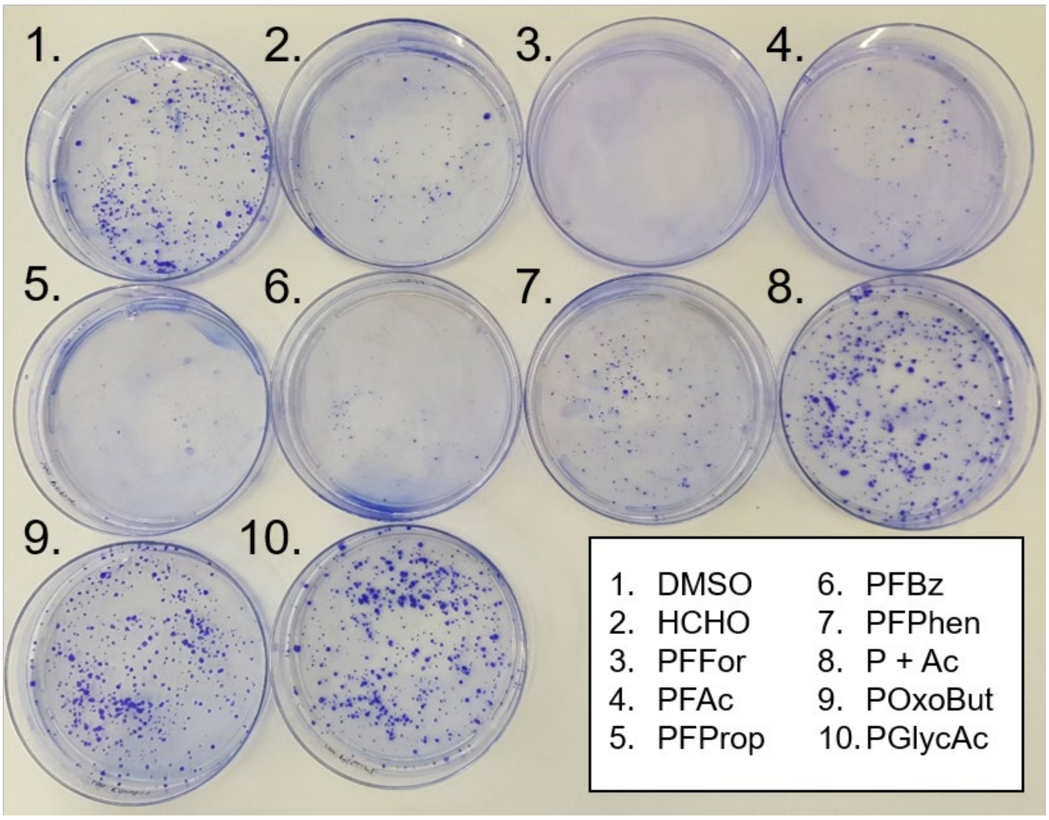

Figure S11. Images of petri dishes seeded with U2OS cells (1000 cells per dish) and treated with either DMSO (1.), HCHO (50 µM, 2.), PFFor (100 µM, 3.), PFAc (100 µM, 4.), PFProp (100 µM, 5.), PFBz (100 µM, 6.), PFPhen (100 µM, 7.), phthalimide and acetic acid (P + Ac, 1:1 ratio, 100 µM each, 8.), POxoBut (100 µM, 9.) or PGlycAc (100 µM, 10.). Growth inhibition appears dependent on HCHO release.

Table S1. Raw abundance values for hydroxymethyl-dimedone and thymidine derivatives from the metabolomics analyses. Five biological replicates were analysed for each experiment.

Hydroxymethyl-dimedone	No Treatment	572004	639031	591703	562875	526386
	HCHO	673718	633371	757814	686863	776508
	PFAc	1564428	1264816	1118648	874118	800817
dTMP	No Treatment	15.4983	32.8309	27.0659	30.7993	30.9213
	HCHO	35.7811	28.8408	31.4700	27.1712	34.7035
	PFAc	337.7858	118.7326	99.6714	57.4018	47.8768
dTDP	No Treatment	125.7121	203.8631	227.4609	210.3804	209.2005
	HCHO	232.0226	232.4495	188.8582	244.3846	294.6830
	PFAc	3900.7004	998.1550	692.2907	376.0631	296.1212
dTTP	No Treatment	1778.4619	3150.5701	2765.2381	2302.1410	2337.1144
	HCHO	2673.9787	2983.0517	1987.0797	2996.1120	2959.0241
	PFAc	22240.9388	7089.1057	4561.2553	3613.4652	3018.5334

Table S2. DPC ratios calculated for each replicate from the DPC detection analyses. With the exception of P + F, experiments were conducted on at least two separate occasions.

No Treatment	0.136532	0.139991	0.159550	0.174870	0.171645	
DMSO	0.149686	0.144853	0.133053	0.116764	0.222071	0.181871
HCHO	0.486030	0.287965	0.443890	0.485225	0.393404	0.378414
PGlycAc	0.100120	0.120005	0.130994	0.133922		
POxoBut	0.217916	0.141473	0.117191	0.137650		
P	0.141453	0.127220	0.099402	0.175964		
P + F	0.137833	0.101853				
PFAc	0.222177	0.222239	0.195981	0.232839	0.156918	0.168386
PFFor	0.424386	0.372336	0.332765	0.276745		

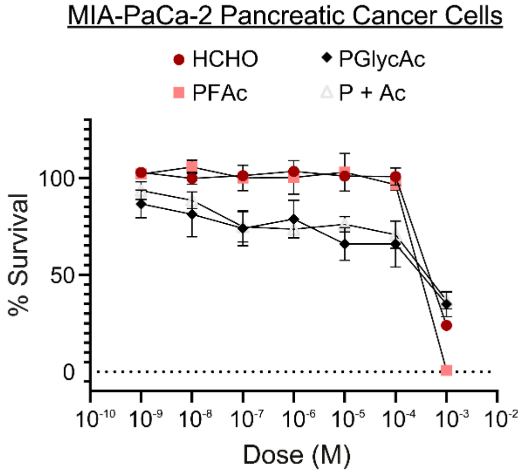

Figure S12. MTT cytotoxicity assays showing concentration-dependent toxicity of HCHO, PFAc, PGlycAc and P + C in MIA-PaCa-2 pancreatic cancer cells. Significant toxicity was only observed at 1 mM in all samples. Error bars represent standard deviations (n = 3).

<u>**Chemical Synthesis and Characterisation**</u>

(1,3-Dioxoisoindolin-2-yl)methyl formate (PFFor)

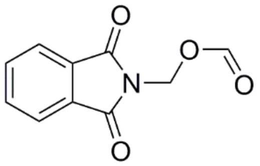

Yield = 44 %. mp 99.7 – 100.6 °C. IR υ(cm^{-1}) 1718, 2990. ^{1}H NMR (500 MHz, DMSO-d_8) δ(ppm) 5.68 (s, 2H, NCH_2O), 7.90-8.01 (m, 4H, ArCH), 8.32 (s, 1H, CHO). ^{13}C NMR (125 MHz, DMSO-d_8) δ(ppm) 60.7, 124.2, 131.8, 135.6, 161.9, 167.0. HRMS (ES+) $C_{10}H7NO_4Na$ $[M+Na]^+$ requires 228.0273 found 228.0275. IR V_{max} (FTIR/neat cm-1) 1777 (C=O), 1723 (C=O), 1223 (C-O), m.p. 99.7 – 100.6 °C.

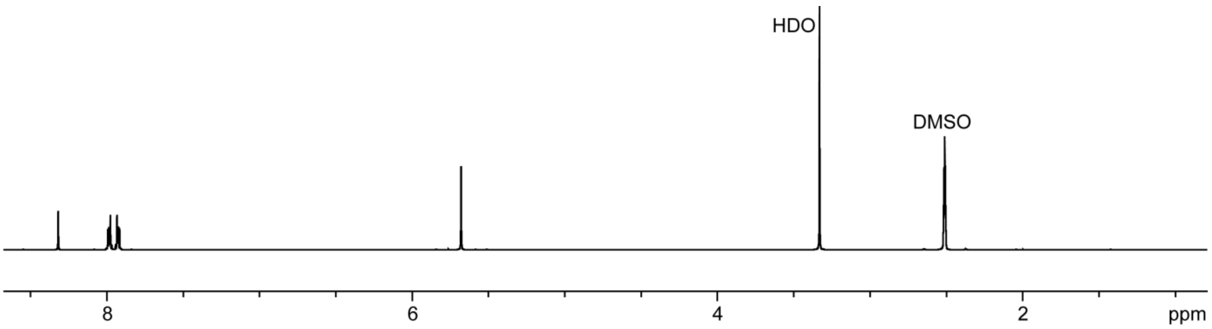

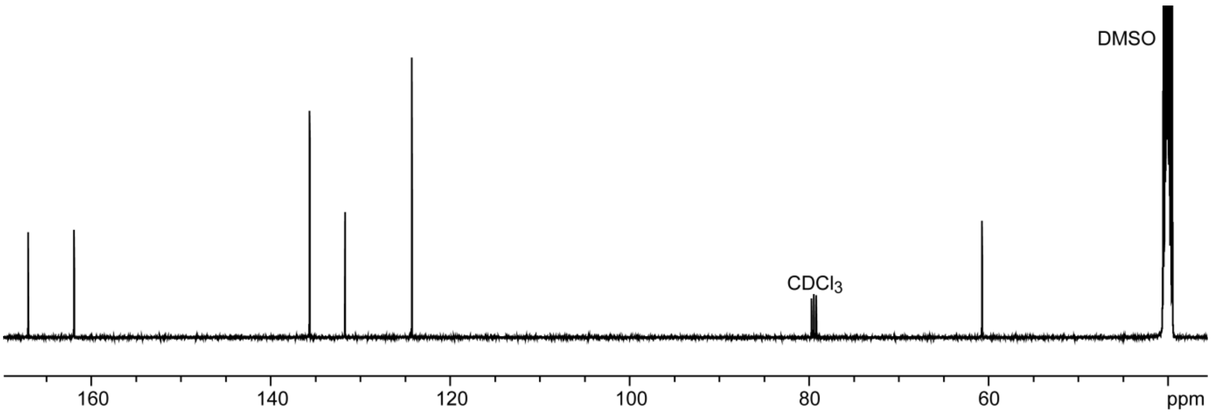

(1,3-Dioxoisoindolin-2-yl)methyl acetate (PFAc)

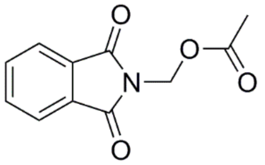

Yield = 48 %. mp 110.4 – 111.6 °C. IR υ(cm^{-1}) 1722, 2964. ^{1}H NMR (500 MHz, DMSO-d_8) δ(ppm) 2.04 (s, 3H, CH_3), 5.58 (s, 2H, NCH_2O), 7.89-8.00 (m, 4H, ArCH). ^{13}C NMR (125 MHz, DMSO-d_8) δ(ppm) 21.0, 60.9, 124.2, 131.7, 135.6, 167.0, 169.9. HRMS (ES+) $C_{11}H_9NO_4Na$ $[M+Na]^+$ requires 242.04290 found 242.0433. IR V_{max} (FTIR/neat cm-1) 1777 (C=O), 1724 (C=O), 1223 (C-O), m.p. 110.4 – 111.6 °C.

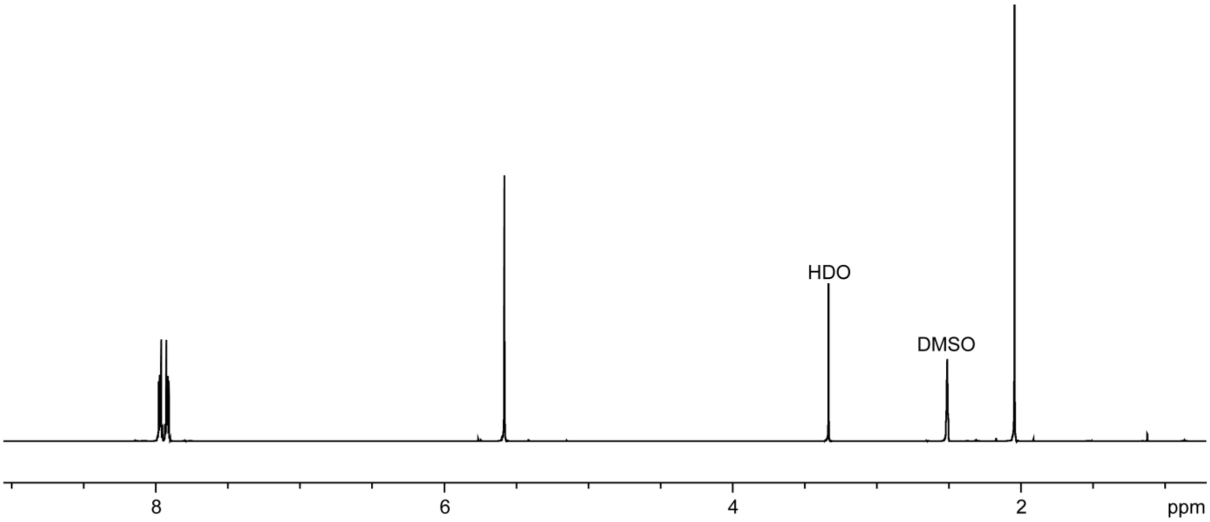

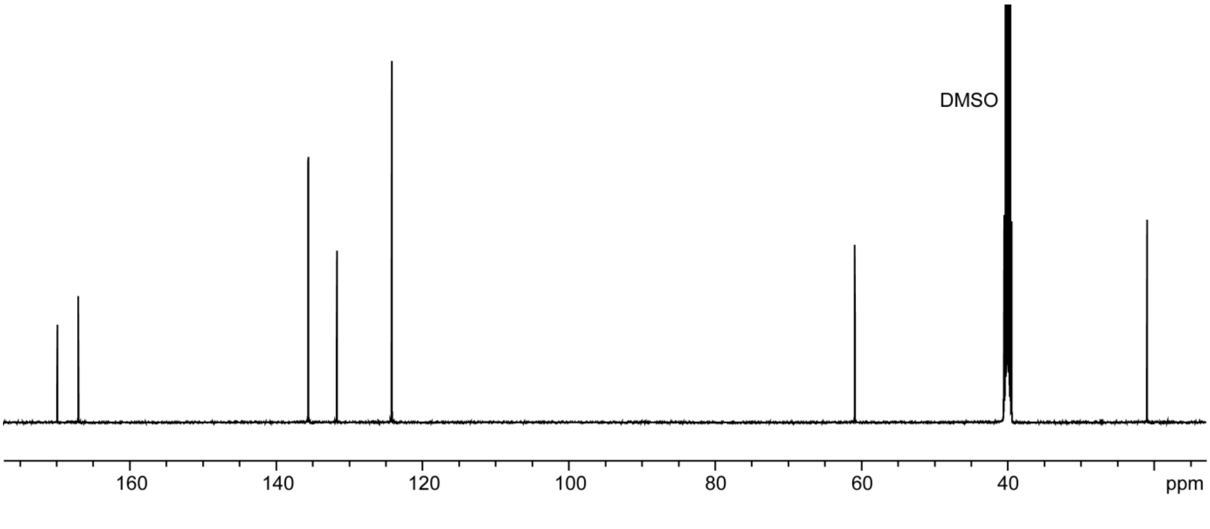

(1,3-Dioxoisoindolin-2-yl)methyl propionate (PFProp)

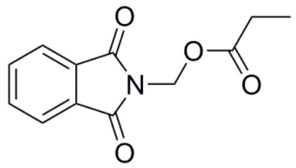

Yield = 27 %. mp 82.8 – 83.4 °C. IR υ(cm^{-1}) 1716, 2980. ^{1}H NMR (400 MHz, $CDCl_3$) δ(ppm) 1.07 (t, *J* = 7.5 Hz, 3H, CH_3), 2.28 (q, *J* = 7.5 Hz, 2H, $CH_3C\underline{\mathit{H}_2}$), 5.66 (s, 2H, NCH_2O), 7.69-7.75 (m, 2H, ArCH), 7.84-7.89 (m, 2H, ArCH). ^{13}C NMR (101 MHz, $CDCl_3$) δ(ppm) 8.8, 27.2, 60.8, 124.0, 131.7, 134.6, 166.8, 173.3. HRMS (ES+) $C_{12}H_{11}NO_4Na$ $[M+Na]^+$ requires 256.0586 found 256.0589. IR v_{max} (FTIR/neat cm^{-1}) 1783 (C=O), 1715 (C=O), 1176 (C-O) m.p. 82.8 – 83.4 °C.

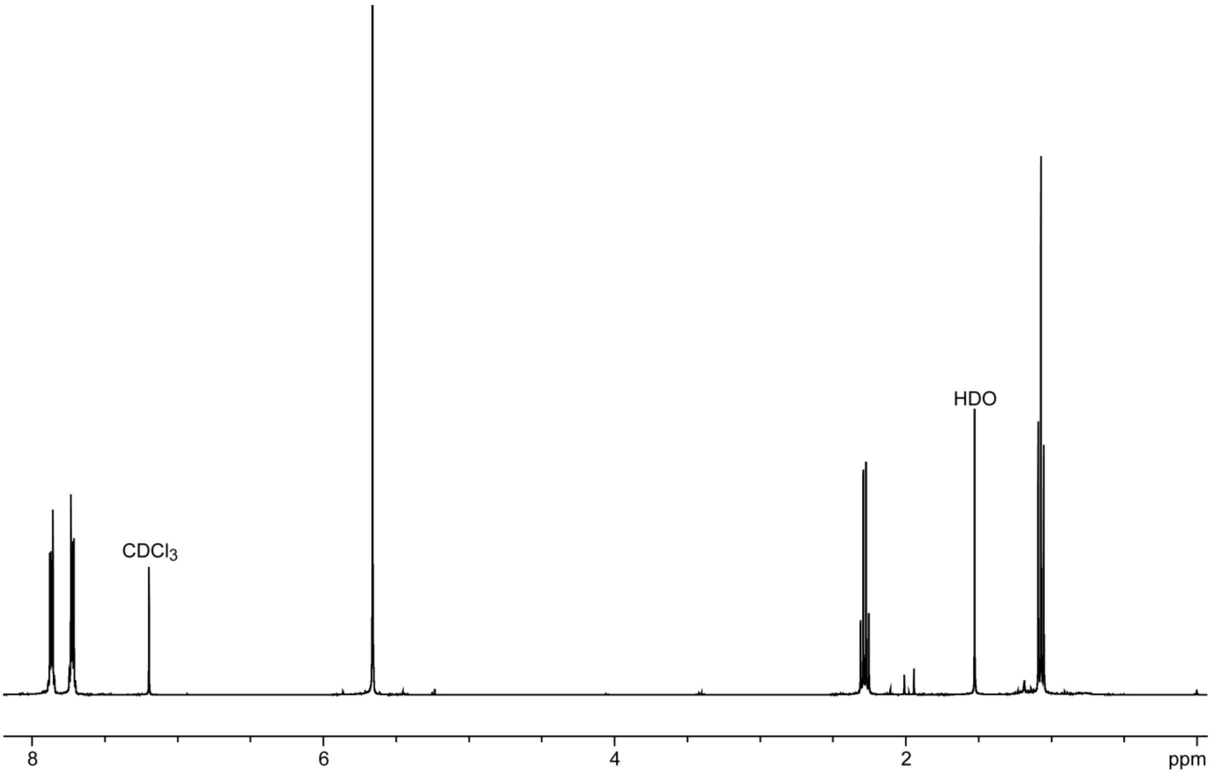

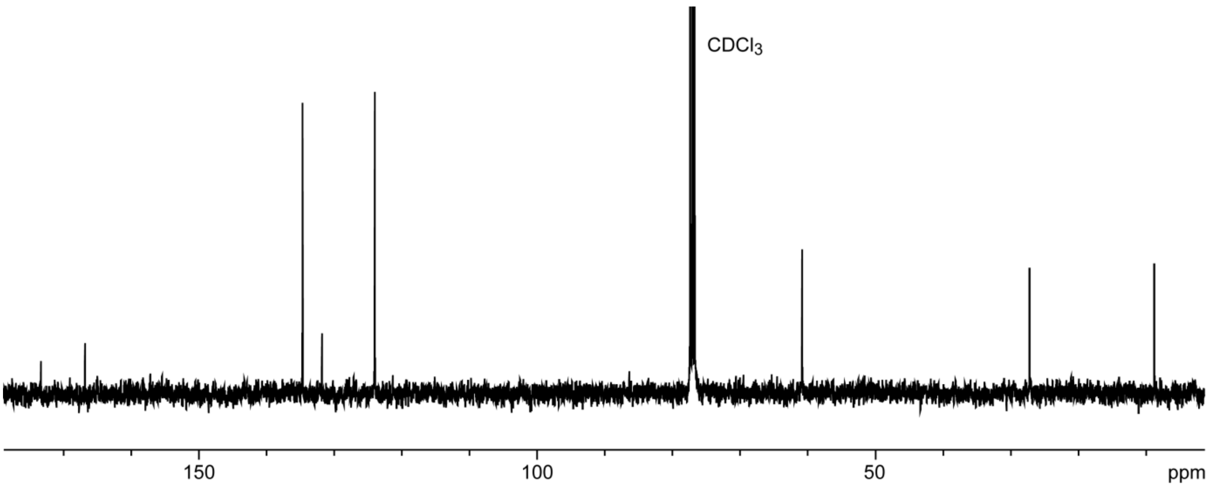

(1,3-Dioxoisoindolin-2-yl)methyl pivalate (PFPiv)

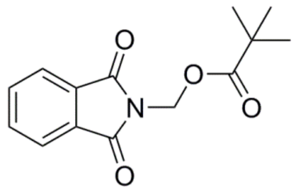

Yield = 51 %. mp 98.8 – 102.3 °C. IR υ(cm^{-1}) 1726, 2978. ^{1}H NMR (500 MHz, DMSO-d_8) δ(ppm) 1.12 (s, 9H, CH_3), 5.60 (s, 2H, NCH_2O), 7.89-8.00 (m, 4H, ArCH). ^{13}C NMR (125 MHz, DMSO-d_8) δ(ppm) 27.1, 38.8, 61.4, 124.2, 131.7, 135.6, 167.0, 176.9. HRMS (ES+) $C_{14}H_{15}NO_4Na$ $[M+Na]^+$ requires 284.0899 found 284.0899. IR v_{max} (FTIR/neat cm^{-1}) 1777 (C=O), 1726 (C=O), 1133 (C-O), m.p. 98.8 – 102.3 °C.

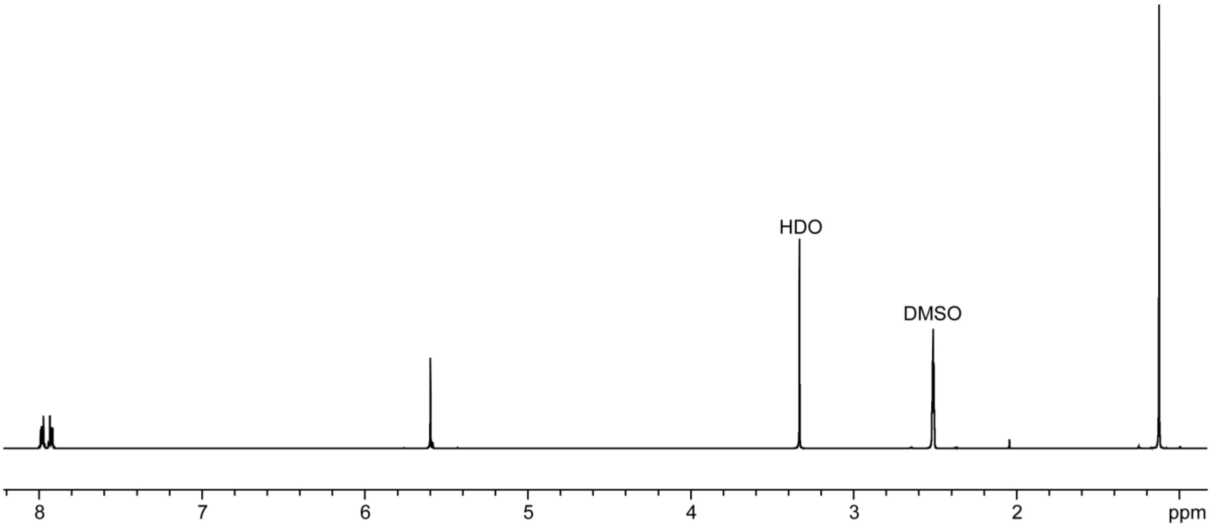

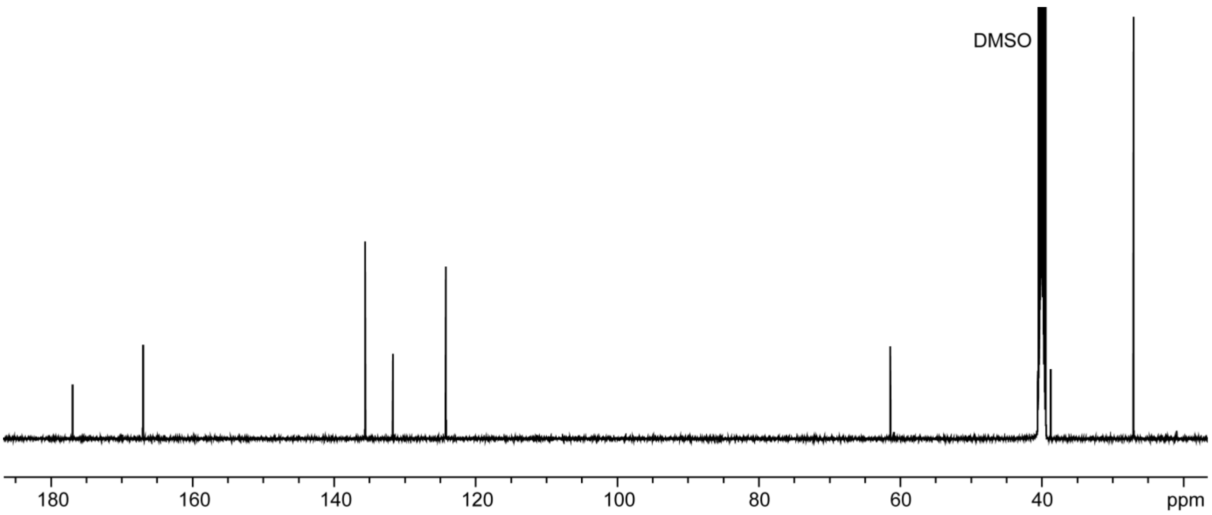

(1,3-Dioxoisoindolin-2-yl)methyl 3-methylpentanoate (PF3MPent)

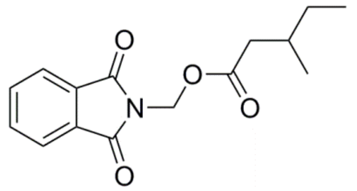

Yield = 47 %. mp 39.6 – 40.8 °C. IR υ(cm^{-1}) 1721, 2960. ^1H NMR (400 MHz, $CDCl_3$) δ(ppm) 0.79 (t, J = 7.5 Hz, 3H, $CH_2C\underline{H_3}$), 0.85 (d, J = 6.5 Hz, 3H, $CHC\underline{H_3}$), 1.08-1.34 (m, 2H, $C\underline{H}_2CH_3$), 1.74-1.87 (m, 1H, $C\underline{H}CH_3$), 2.04 (dd, J_1 = 8.0 Hz, J_2 = 15 Hz, 1H, $C\underline{H}_2CH$), 2.26 (dd, J_1 = 6.0 Hz, J_2 = 15 Hz, 1H, $C\underline{H}_2CH$), 5.65 (s, 2H, NCH_2O), 7.69-7.75 (m, 2H ArCH), 7.83-7.89 (m, 2H, ArCH). ^{13}C NMR (101 MHz, $CDCl_3$) δ(ppm) 11.2, 19.2, 29.3, 31.7, 41.0, 60.7, 124.0, 131.8, 134.6, 166.8, 172.2. HRMS (ES+) $C_{15}H_{17}NO_4Na$ $[M+Na]^+$ requires 298.1055 found 298.1058. IR V_{max} (FTIR/neat cm-1) 2957 (C-H), 2854 (C-H), 1777 (C=O), 1722 (C=O), 1182 (C-O), m.p. 39.6 – 40.8 °C.

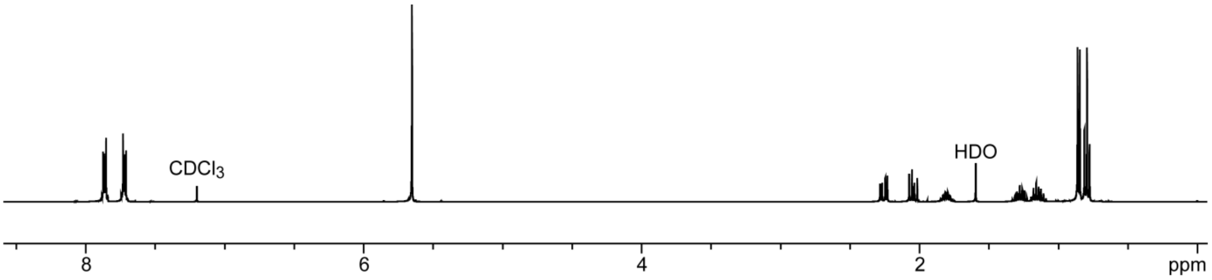

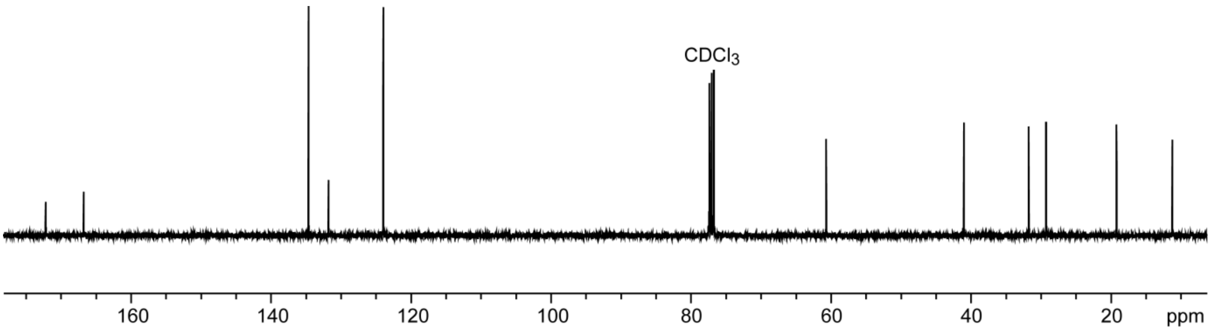

(1,3-Dioxoisoindolin-2-yl)methyl benzoate (PFPhen)

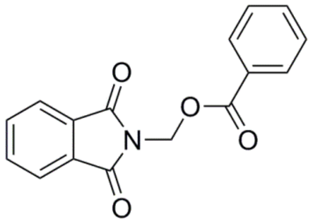

Yield = 34 %. mp 104.6 – 100.6 °C. IR υ(cm^{-1}) 1716. ^1H NMR (500 MHz, DMSO-d_8) δ(ppm) 5.87 (s, 2H, NCH_2O), 7.53 (app t, *J* = 8.0 Hz, 2H, BzCH), 7.68 (tt, J_1 = 1.5 Hz, J_2 = 7.5 Hz, 1H, BzCH), 7.90-8.03 (m, 6H, ArCH, BzCH). ^{13}C NMR (125 MHz, DMSO-d_8) δ(ppm) 61.8, 124.2, 129.2, 129.3, 129.9, 131.8, 134.3, 135.6, 165.2, 167.1. HRMS (ES+) $C_{16}H_{11}NO_4Na$ $[M+Na]^+$ requires 304.0586 found 304.0589. IR V_{max} (FTIR/neat cm-1) 1781 (C=O), 1715 (C=O), 1260 (C-O), m.p. 104.6 – 105.2 °C.

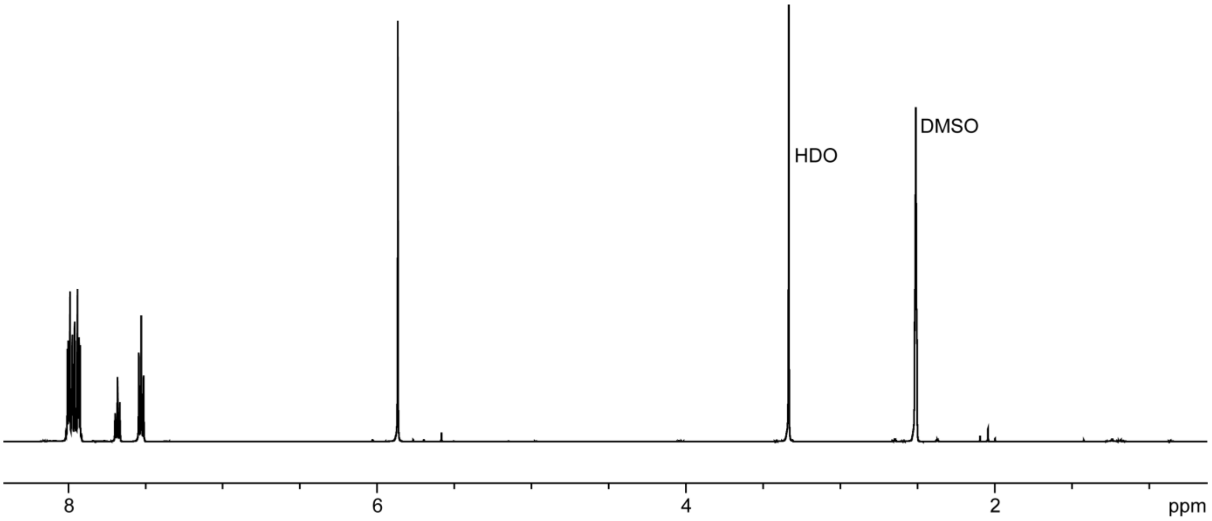

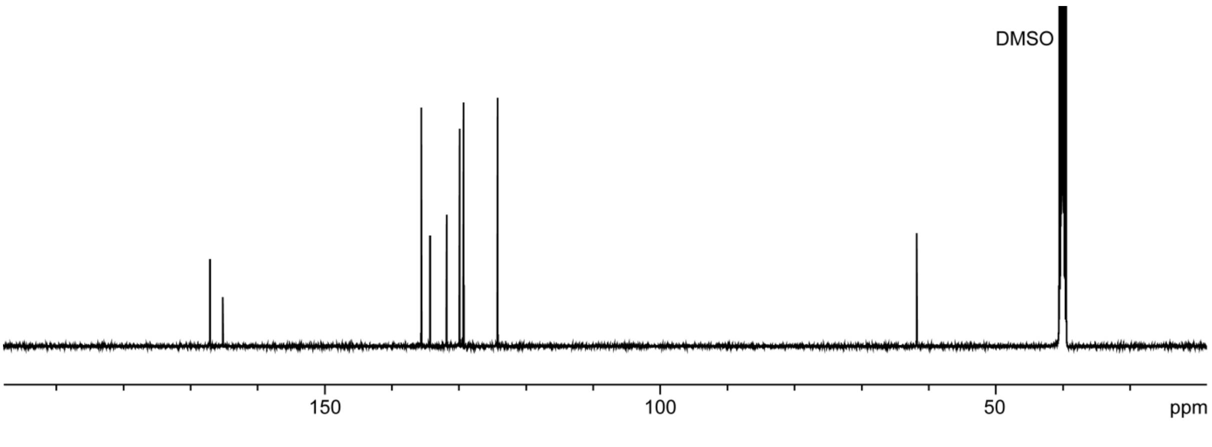

(1,3-Dioxoisoindolin-2-yl)methyl 2-phenylacetate (PFBz)

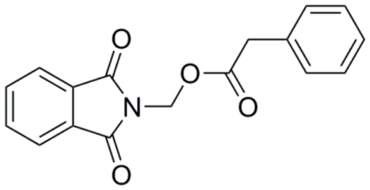

Yield = 63 %. mp 107.5 – 108.6 °C. IR υ(cm^{-1}) 1724, 2924. ^{1}H NMR (400 MHz, $CDCl_3$) δ(ppm) 3.57 (s, 2H, C*H*$_2$Ph), 5.67 (s, 2H, NCH_2O), 7.15-7.26 (m, 5H, Ph), 7.68-7.75 (m, 2H, ArCH), 7.82-7.89 (m, 2H, ArCH). ^{13}C NMR (101 MHz, $CDCl_3$) δ(ppm) 40.8, 61.2, 124.0, 127.3, 128.6, 129.3, 131.7, 133.2, 134.7, 166.7, 170.5. HRMS (ES+) $C_{17}H_{13}NO_4Na$ $[M+Na]^+$ requires 318.0742 found 318.0743. IR V_{max} (FTIR/neat cm-1) 2920 (C-H), 2850 (C-H), 1777 (C=O), 1724 (C=O), 1229 (C-O), m.p. 107.5 – 108.6 °C.

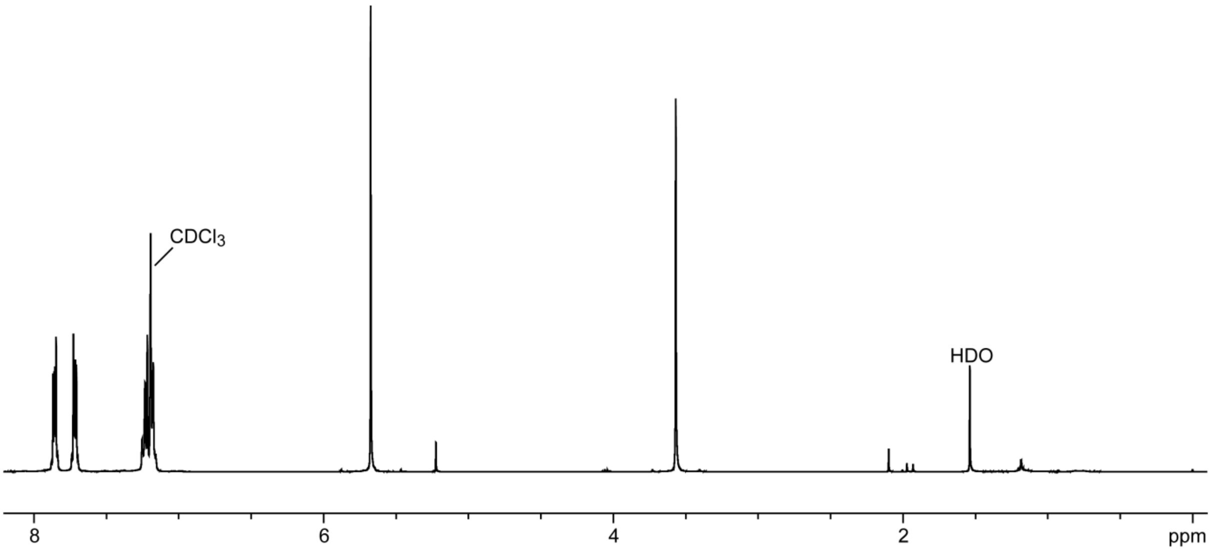

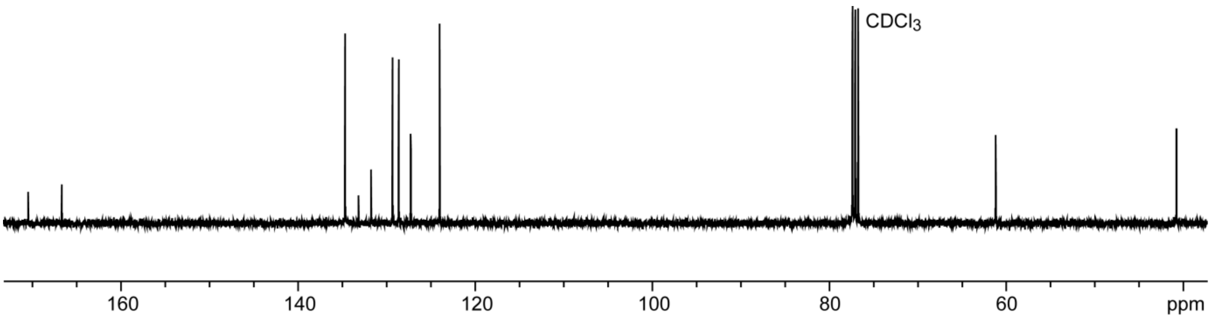

(1,3-Dioxoisoindolin-2-yl)methyl oleate (PFOle)

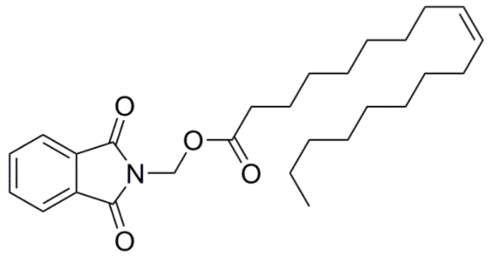

Yield = 69 %. mp 28.9 – 30.4 °C. IR υ(cm^{-1}) 1717, 2918. ^{1}H NMR (500 MHz, DMSO-d$_8$) δ(ppm) 0.84 (t, *J* = 7.0 Hz, 3H, CH_3), 1.15-1.33 (m, 20H, 10×CH_2), 1.45-1.55 (m, 2H, $COCH_2C\underline{H_2}$), 1.88-2.02 (m, 4H, $C\underline{H_2}CH=CHC\underline{H_2}$), 2.31 (t, *J* = 7.5 Hz, 2H, $COCH_2$), 5.26-5.35 (m, 2H, CH=CH), 5.60 (s, 2H, NCH_2O), 7.89-8.00 (m, 4H, ArCH). ^{13}C NMR (125 MHz, DMSO-d$_8$) δ(ppm) 14.4, 22.6, 24.7, 26.9, 27.0, 28.7, 28.8, 28.9, 29.0, 29.1, 29.3, 29.4, 29.5, 31.7, 33.6, 60.8, 124.2, 130.0, 130.1, 131.7, 135.6, 167.0, 172.4. HRMS (ES+) $C_{27}H_{39}NO_4Na$ $[M+Na]^+$ requires 464.2777 found 464.2773. IR V_{max} (FTIR/neat cm-1) 2918 (C-H), 2850 (C-H), 1748 (C=O), 1715 (C=O) 1192 (C-O), m.p. 28.9 – 30.4 °C.

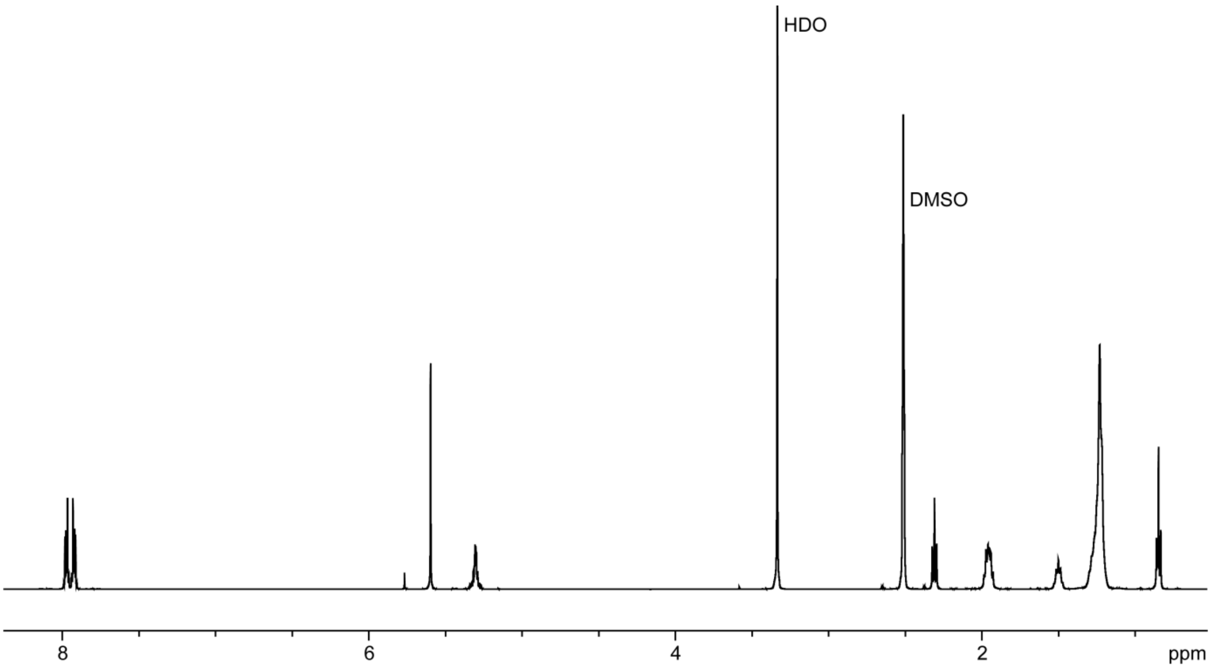

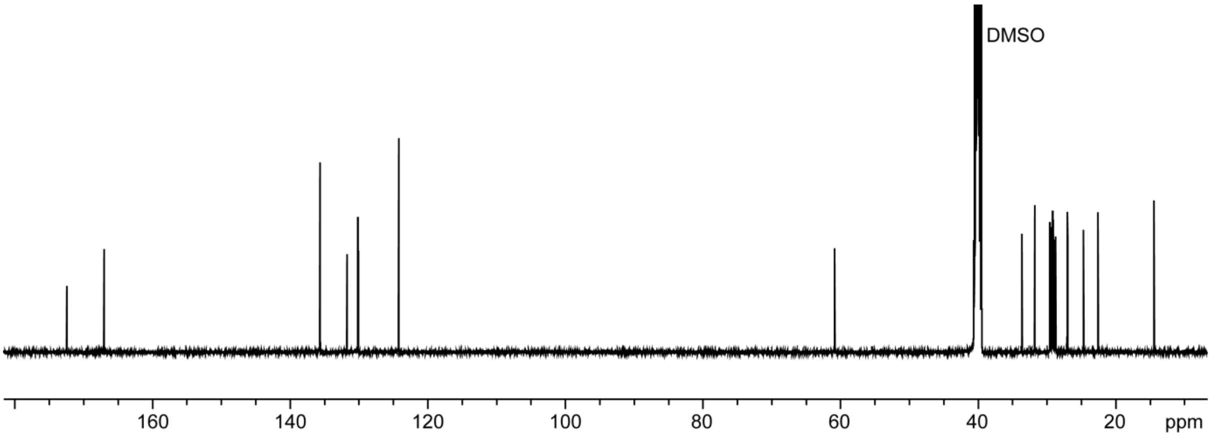

Bis((1,3-dioxoisoindolin-2-yl)methyl) succinate (PFSucc)

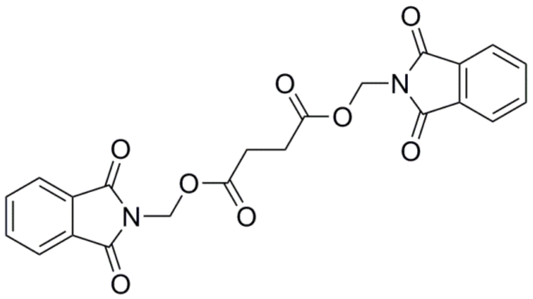

Yield = 68 %. mp 194.4 – 196.1 °C. IR υ(cm^{-1}) 1720, 2979.^{1}H NMR (500 MHz, DMSO-d_8) δ(ppm) 2.59 (s, 4H, CH_2CH_2), 5.58 (s, 4H, NCH_2O), 7.89-8.00 (m, 8H, ArCH). ^{13}C NMR (125 MHz, DMSO-d_8) δ(ppm) 28.6, 61.1, 124.2, 131.7, 135.6, 137.0, 171.4. HRMS (ES+) $C_{22}H_{16}N_2O_8Na$ [M+Na]$^{+}$ requires 459.0804 found 459.0800. IR v_{max} (FTIR/neat cm^{-1}) 1777 (C=O), 1748 (C=O), 1720 (C=O), 1233 (C-O), m.p. 194.4 – 196.1 °C.

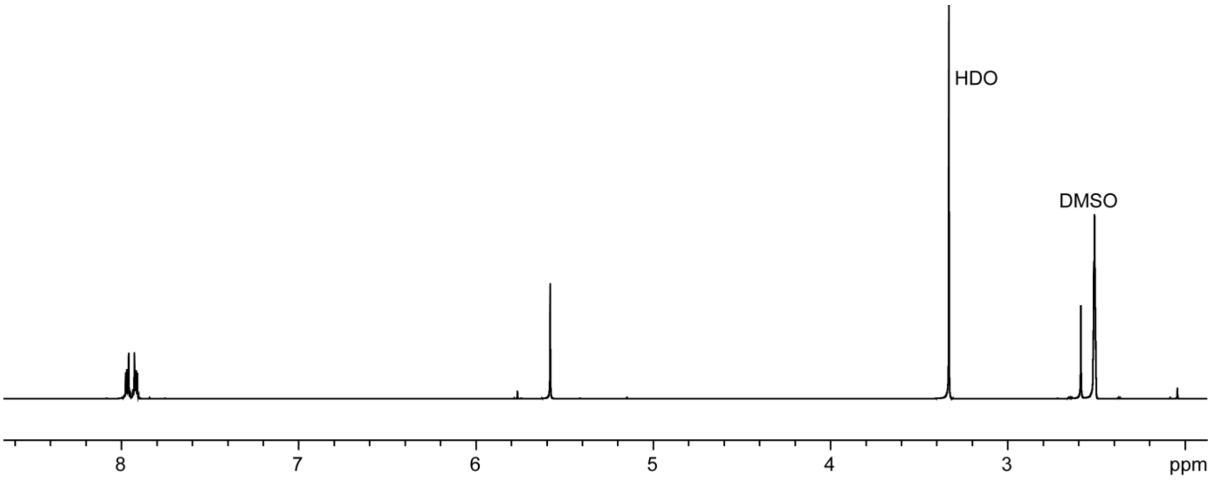

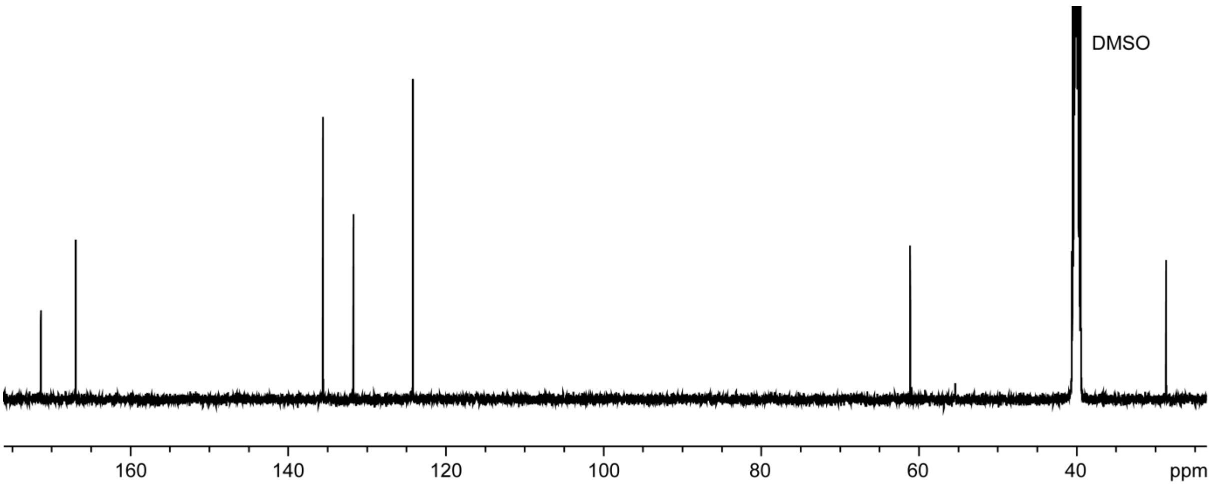

2-(3-Oxobutyl)isoindoline-1,3-dione (POxoBut)

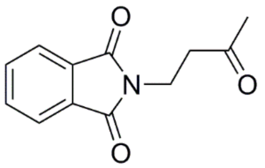

Phthalimide (147 mg, 1 mmol) was mixed with 3-buten-2-one (59 µL, 1.1 mmol) in EtOH (5 mL) in a microwave reaction vial. NaOEt (75 mg, 1.1 mmol) was then added. The reaction vial was sealed and stirred for 12 hours and room temperature. The vial was then heated at 100 °C for 1 hour in a microwave reactor (Biotage) before the solvent was removed *in vacuo* and the product purified by Flash chromatography (conditions: 95 % CH_2Cl_2, 5 % MeOH, 10 CV). The product was isolated after evaporation of the solvent *in vacuo*. Yield = 20 %. mp 107.4 – 108.5 °C. IR υ(cm^{-1}) 1715, 2935. ^{1}H NMR (500 MHz, DMSO-d_8) δ(ppm) 2.12 (s, 3H, CH_3), 2.84 (t, *J* = 7.5 Hz, 2H, CH_2CO), 3.77 (t, *J* = 7.5 Hz, 3H, NCH_2), 7.82-7.90 (m, 4H, ArCH). ^{13}C NMR (125 MHz, DMSO-d_8) δ(ppm) 30.2, 33.1, 41.4, 123.5, 132.1, 134.9, 168.2, 207.0. HRMS (ES+) $C_{12}H_{12}NO_3$ $[M+H]^+$ requires 218.0817 found 218.0817. IR v_{max} (FTIR/neat cm^{-1}) 1769 (C=O), 1715 (C=O), 1699 (C=O), 1174 (C-O), m.p. 107.4 – 108.5 °C.

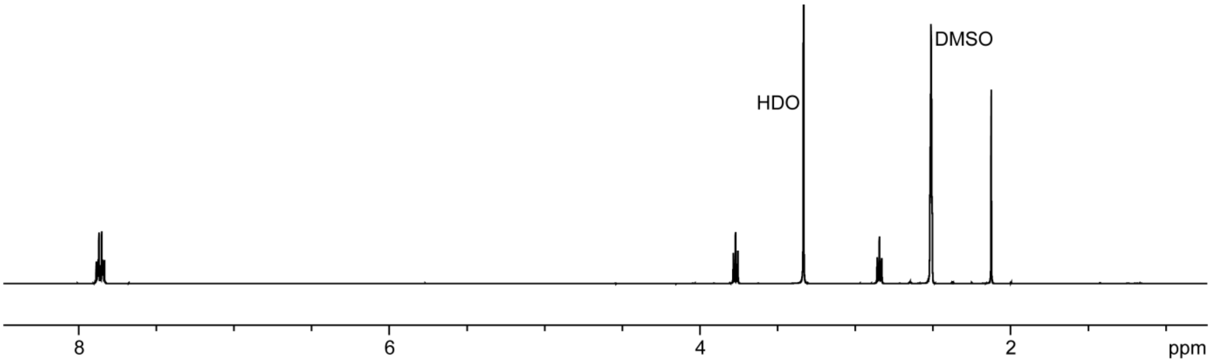

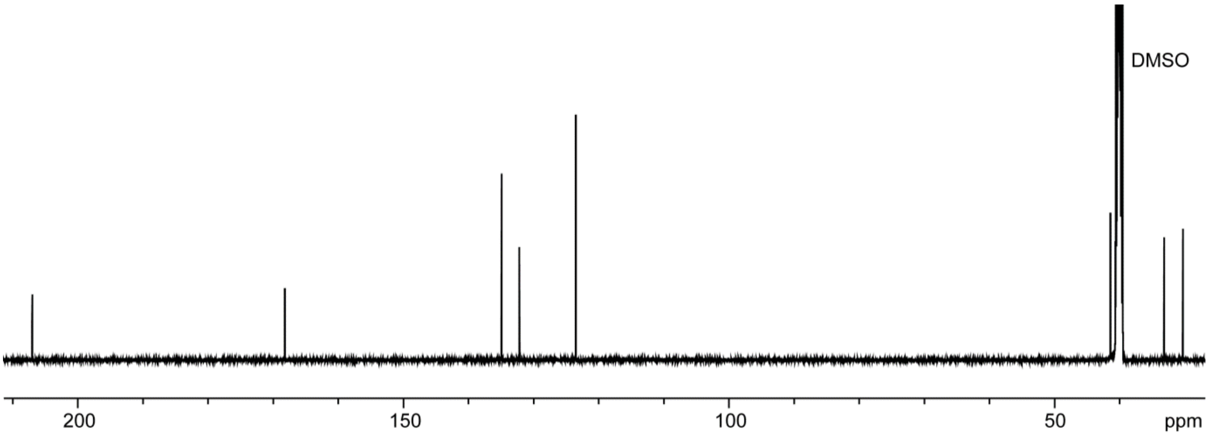

2-(1,3-Dioxoisoindolin-2-yl)ethyl acetate (PGlycAc)

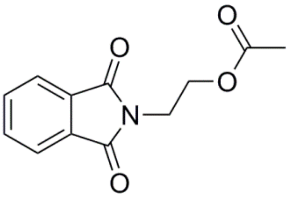

(*N*-hydroxyethyl)-phthalimide (191 mg, 1 mmol) and acetyl chloride (32 µL, 1.1 mmol) were mixed in EtOAc (5 mL) in a vial before DIPEA (192 µL, 1.1 mmol) was added dropwise. The reaction was then stirred for 12 hours at room temperature. The product was isolated by removal of the solvent *in vacuo* and purified by Flash chromatography (conditions: 95 % CH_2Cl_2, 5 % MeOH, 10 CV). The product was isolated after evaporation of the solvent *in vacuo*. Yield = 33 %. mp 87.9 – 88.3 °C. IR υ(cm^{-1}) 1707, 2980. ^{1}H NMR (500 MHz, DMSO-d_8) δ(ppm) 1.94 (s, 1H, CH_3), 3.83 (t, *J* = 5.5 Hz, 2H, CH_2N), 4.23 (t, *J* = 5.5 Hz, 2H, CH_2O), 7.84-7.92 (m, 4H, ArCH). NMR (125 MHz, DMSO-d_8) δ(ppm) 21.0, 37.3, 65.4, 123.6, 132.0, 135.0, 168.2, 170.8. HRMS (ES+) $C_{12}H_{11}NO_4Na$ [M+Na]$^+$ requires 256.0586 found 256.0587. IR v_{max} (FTIR/neat cm^{-1}) 1736 (C=O), 1705 (C=O), 1238 (C-O), m.p. 87.9 – 88.3 °C.

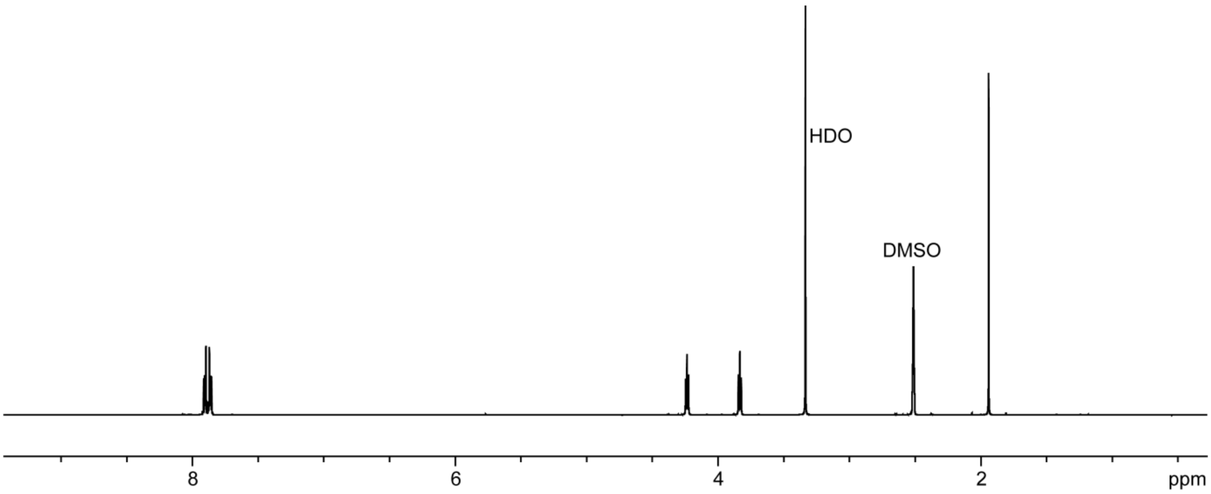

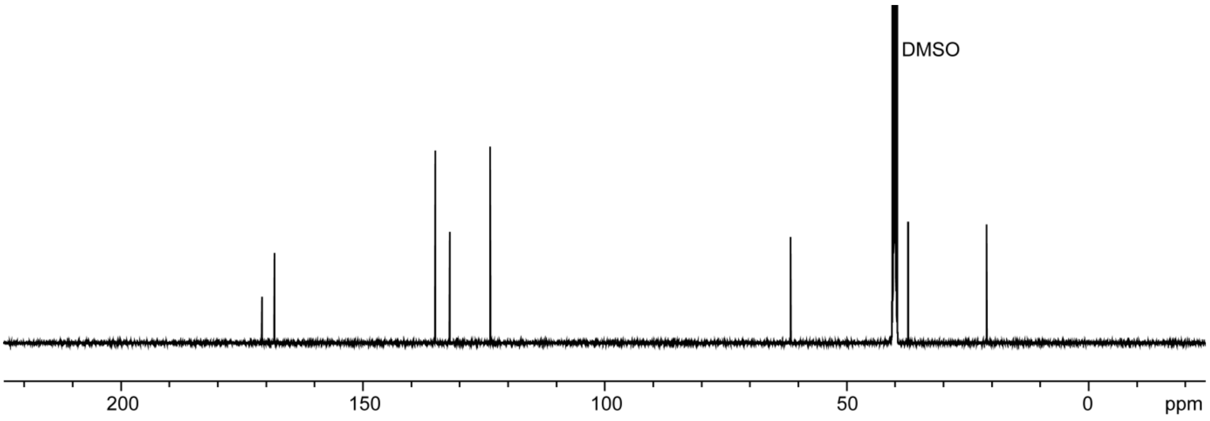

Supplementary References

1 J. Walsby-Tickle, J. Gannon, I. Hvinden, C. Bardella, M. I. Abboud, A. Nazeer, D. Hauton, E. Pires, T. Cadoux-Hudson, C. J. Schofield and J. S. O. McCullagh, *Commun. Biol.*, 2020, **3**, 247.

2 A. Zhitkovich and M. Costa, *Carcinogenesis*, 1992, **13**, 1485-1489.
